# Applications of Carbon-Based Multivariable Chemical Sensors for Analyte Recognition

**DOI:** 10.1007/s40820-025-01741-0

**Published:** 2025-05-03

**Authors:** Lin Shi, Jian Song, Yu Wang, Heng Fu, Kingsley Patrick-Iwuanyanwu, Lei Zhang, Charles H. Lawrie, Jianhua Zhang

**Affiliations:** 1 School of Microelectronics, Shanghai University, Shanghai, 201800 People’s Republic of China; 2 Sino-Swiss Institute of Advanced Technology (SSIAT), Shanghai University, Shanghai, 201899 People’s Republic of China; 3 Department of Biochemistry (Toxicological Unit), University of Port Harcourt, Port Harcourt, Nigeria; 4 Biogipuzkoa Health Research Institute, San Sebastian, 20014 Spain; 5 IKERBASQUE, Basque Foundation for Science, Bilbao, 48009 Spain; 6 Radcliffe Department of Medicine, University of Oxford, Oxford, OX3 9DU UK

**Keywords:** Carbon nanotubes, Graphene, Field-effect transistors, Gas sensors, Biosensors

## Abstract

Over recent decades, carbon-based chemical sensor technologies have advanced significantly. Nevertheless, significant opportunities persist for enhancing analyte recognition capabilities, particularly in complex environments. Conventional monovariable sensors exhibit inherent limitations, such as susceptibility to interference from coexisting analytes, which results in response overlap. Although sensor arrays, through modification of multiple sensing materials, offer a potential solution for analyte recognition, their practical applications are constrained by intricate material modification processes. In this context, multivariable chemical sensors have emerged as a promising alternative, enabling the generation of multiple outputs to construct a comprehensive sensing space for analyte recognition, while utilizing a single sensing material. Among various carbon-based materials, carbon nanotubes (CNTs) and graphene have emerged as ideal candidates for constructing high-performance chemical sensors, owing to their well-established batch fabrication processes, superior electrical properties, and outstanding sensing capabilities. This review examines the progress of carbon-based multivariable chemical sensors, focusing on CNTs/graphene as sensing materials and field-effect transistors as transducers for analyte recognition. The discussion encompasses fundamental aspects of these sensors, including sensing materials, sensor architectures, performance metrics, pattern recognition algorithms, and multivariable sensing mechanism. Furthermore, the review highlights innovative multivariable extraction schemes and their practical applications when integrated with advanced pattern recognition algorithms.

## Highlights

- This paper reviews the fundamentals and research progress of carbon-based multivariable chemical sensors, with a particular focus on the classification and identification of multiple analytes.
- Carbon-based multivariable chemical sensors consisting of carbon nanotubes/graphene as the sensing material and field effect transistors as the transducers are discussed in detail.
- A comprehensive analysis of multivariable sensing mechanisms is presented and design criteria for carbon-based multivariable sensors are summarized.

## Graphical abstract

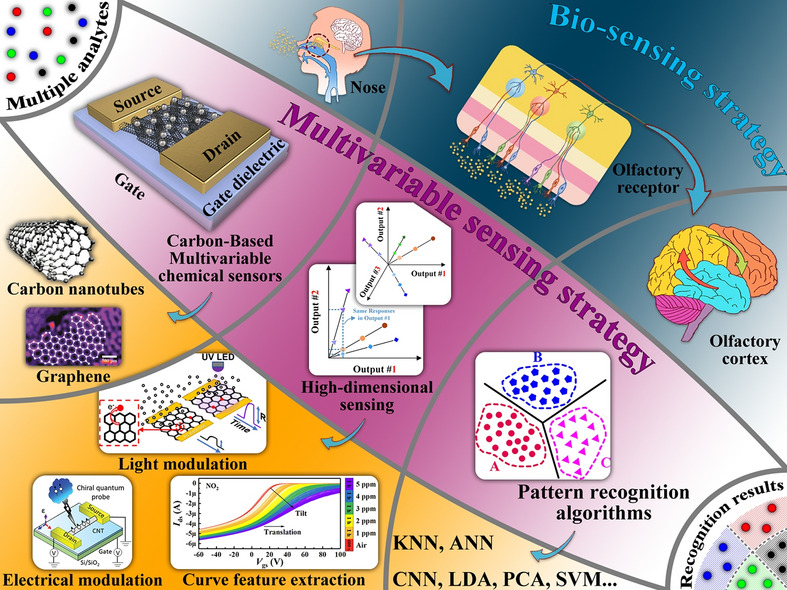

## Introduction

In recent years, chemical sensors have been widely employed for environmental monitoring, industrial production, and medical diagnostics [1–6]. There are two major test prerequisites for the practical application of sensors within complex chemical environments. Firstly, the accurate measurement of one component, and secondly, the classification and identification of multiple or all chemical components. In order to meet the former requirement, it is necessary for the sensor to demonstrate a high degree of selectivity, that is, to be resistant to interference from other analytes present in the environment. In order to satisfy the second requirement, the sensor must demonstrate a differentiated response to a range of analytes and possess the capacity to incorporate pattern recognition algorithms for the identification of analyte species and concentrations. While gas chromatograph, mass spectrometer, and high-performance liquid chromatography are capable of fulfilling both of these testing needs, their considerable size and intricate operational requirements restrict their deployment in portable, real-time monitoring applications [7].

With the development of the Internet of Things (IoT) and the industrial internet, there is a growing demand for low-cost, compact chemical sensors for the construction of sensor networks [8, 9]. Alongside the increasing pursuit of healthy lifestyles, these sensors are expected to facilitate applications such as the detection of harmful substances and the early pre-diagnosis of diseases in home settings [10–12]. Researchers have developed a range of chemical sensors, including optical [13, 14], electrochemical [15, 16], catalytic combustion [17, 18], chemoresistive [19–22], and field-effect transistor (FET) [23, 24]. Catalytic combustion and chemiresistive sensors have achieved commercialization due to their simple manufacturing processes and reliable performance, effectively addressing the challenges of low-cost and large-scale deployment that traditional analytical instruments struggle to overcome in IoT and industrial internet applications [25–27]. Benefiting from advances in microelectronics technology, FET-based chemical sensors can be fabricated at the wafer level using commercially available silicon-based CMOS processes [28–30]. Their microscale dimensions not only enable integration into mobile devices but also provide low-power operation, granting these sensors extended battery life or even self-powered capabilities [31–35]. These advantages effectively compensate for the limitations of traditional analytical instruments in scenarios with space constraints or the absence of external power sources. Furthermore, the integration of microelectronics and biotechnology has facilitated the development of biosensors, enabling users to perform non-invasive, rapid, and real-time monitoring of biomarkers such as microRNA, breast cancer indicators, and SARS-CoV-2 at home [36–38]. These biosensors address the drawbacks of traditional disease detection methods, such as long processing times and invasive procedures, providing an effective solution for early disease detection [39, 40].

**Fig. 1:**
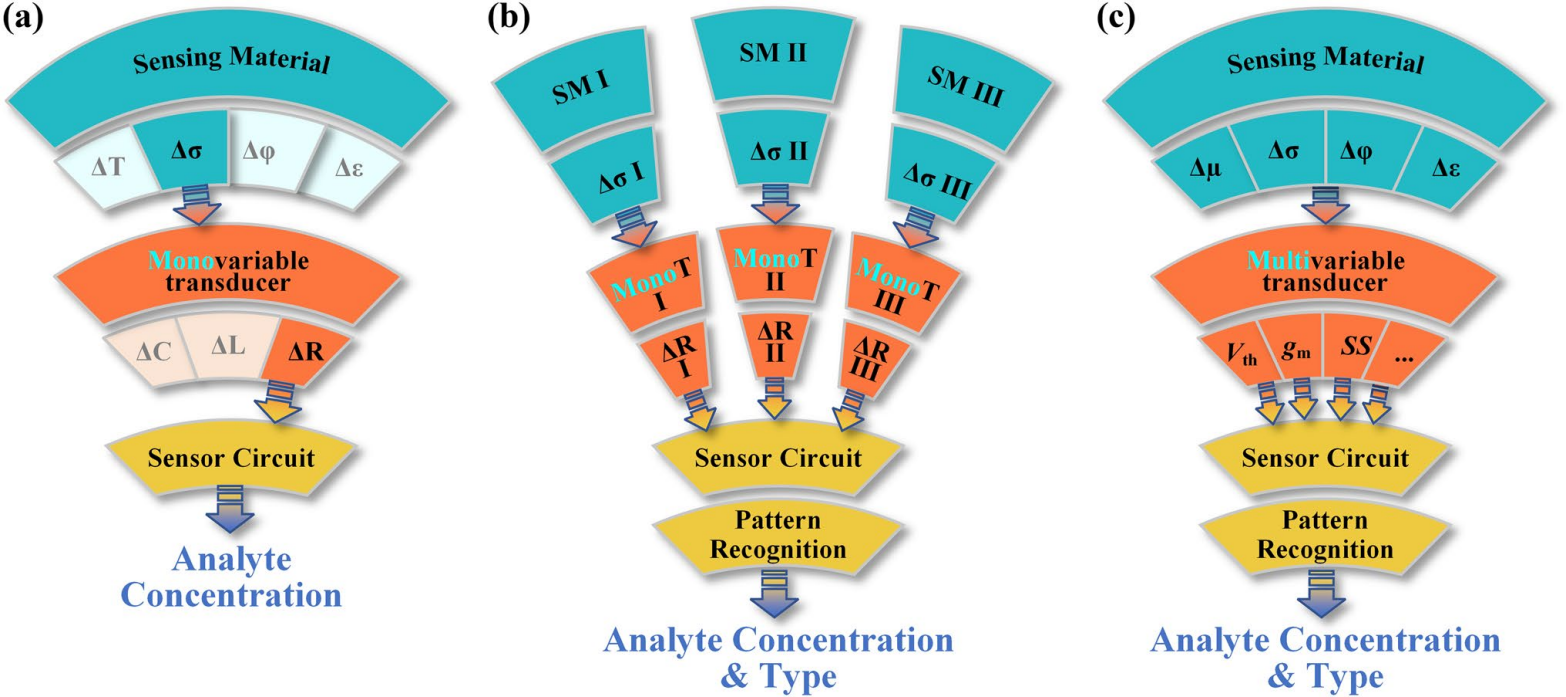
Schematic structures of **a** monovariable sensors, **b** sensor arrays and **c** multivariable sensors. ΔT: Temperature, Δσ: Conductivity, Δφ: Work function, Δε: Permittivity, ΔC: Capacitance, ΔL: Inductance, ΔR: Resistance, SM: Sensing Material, MonoT: Monovariable Transducer, *V*_th_: Threshold voltage, *g*_m_: Transconductance, *SS*: Subthreshold swing

As illustrated in Fig. 1a, chemical sensors are typically composed of two principal components, the sensing material and the transducer [41]. Adsorbing analytes onto sensing materials results in physical property changes such as temperature (ΔT), conductivity (Δσ), work function (Δφ), and permittivity (Δε). These signals are then transformed by transducers into various electrical parameters such as capacitance (ΔC), inductance (ΔL) and resistance (ΔR). Finally these electrical parameters are processed by the sensor circuit to generate an output as analyte concentration information.

Chemoresistive sensors have garnered great interest in recent years due to their simple structure, small size, and good compatibility with sensing materials. In the detection procedure (Fig. 1a), an analyte-induced change in the conductivity (Δσ) of the sensing material is converted by a transducer into a material resistance change (ΔR), which is then processed by the sensor circuit to output analyte concentration information. In this process the sensor’s monovariable transducer only converts a single physical property (Δσ) to a single electrical parameter (ΔR). Despite the analyte’s capability to influence multiple physical properties of the sensing material, the one-to-one conversion results in the loss of a substantial amount of sensing information at the source. In complex chemical environments, the effects of various analytes on sensing materials are mechanically reflected as changes in resistance values, which essentially limit the selectivity and gas identification capabilities of chemoresistive monovariable gas sensors in practical applications. Gardner and Bartlett presented an electronic nose system (sensor array) that was capable of identifying analytes in environments containing a high degree of chemical complexity [42]. Figure 1b illustrates the configuration of the sensor arrays, comprising three sensing units. Each sensing unit has been modified with specific sensing materials (SM), enabling the detection of different analytes (i.e., SM I, SM II, and SM III). The presence of these analytes induces distinctive changes in the physical properties of the sensing materials, as indicated by the corresponding changes in the physical properties (Δσ I, Δσ II, and Δσ III). The aforementioned variations are transformed into electrical parameters (ΔR I, ΔR II, and ΔR III) by the monovariable converters (MonoT I, MonoT II, and MonoT III, MonoT: Monovariable Transducer) of the individual sensing unit. These parameters are subsequently fed into the sensor circuit with pattern recognition algorithms for the determination of the analyte species and concentrations. Although the field of sensor arrays has reached a high level of maturity, its practical limitations are also widely acknowledged. These include the difficulty of data processing due to the uncorrelated drift of each sensing unit, the increase in cost associated with preparing different sensing materials for each sensing unit, and the increase in equipment size due to the continuous expansion in the number of sensing units [8]. In order to achieve analyte identification, sensor arrays employ a methodology that involves the mechanical combination of multiple monovariable sensors, with the objective of increasing the output variable. However, this approach does not fully leverage the potential impact of the analyte on the sensing material. Researchers have developed a number of different types of multivariable chemical sensors, including nonresonant and resonant impedance [43, 44], electromechanical resonant [45, 46], photonic resonant [47, 48], and FET types [49, 50], with the aim of further improving the selectivity and recognition of chemical sensors. Figure 1c illustrates the structure of a FET-type multivariable chemical sensor that has been enhanced with two key improvements. Firstly, the use of sensing materials with multiple response mechanisms to different analytes allows for the acquisition of comprehensive sensing information, including but not limited to Δµ, Δσ, Δφ, and Δε, allowing increased sensing information. Secondly, the output of multiple partially or fully independent electrical parameters (*V*_th_, *g*_m_, and *SS*, etc.) is achieved using multivariable transducers thereby overcoming the issue of a limited number of output variables for monovariable transducers.

In recent years, carbon-based chemical sensors composed of FETs combined with carbon nanotubes (CNTs)/graphene have been the subject of extensive research [51–55]. FETs, as traditional electronic components, possess the characteristics of multivariable output and weak signal amplification. Furthermore, their preparation processes are compatible with modern CMOS processes, which affords them significant potential for application and development [56]. A wide range of high-performance carbon-based materials has emerged in the field of sensing [57–59], including nanoporous carbon with high specific surface area and tunable pore sizes [60, 61], carbon quantum dots with excellent fluorescence properties and biocompatibility [62–64], fullerenes with their unique cage-like structure and electron acceptor capabilities [65, 66], graphene with its two-dimensional planar structure and high carrier mobility [67–69], and CNTs with high aspect ratios and tunable electrical properties [70–72]. Considering the comprehensive sensing performance of these carbon-based materials, graphene and CNTs have found the most widespread applications. In the future roadmap for chemical sensor development [8], advancements in three key directions—high reliability, low cost, and low power consumption—will rely on the integration of sensing materials with advanced micro/nano fabrication technologies to achieve large-scale production [73–75]. The low-dimensional structures and high fabrication efficiency of graphene and CNTs allow for easy large-area deposition on wafers and flexible substrates [76–79]. Additionally, the excellent carrier mobility and semiconductor properties of graphene and CNTs, which are easily adjustable via electrical modulation [80–82], not only facilitate the construction of various electrical transducers but also provide highly sensitive and fast-response detection platforms for chemical sensing. Of particular significance is their ability to provide diverse reaction pathways with multiple analytes, which increases the sensing information obtained in analyte identification.

In the past decade, carbon-based chemical sensors have made remarkable advancements, however, traditional carbon-based chemical sensors utilizing monovariable sensing technology still struggle to compete with commercial sensors, primarily due to poor selectivity and stability. This review provides a detailed analysis of the strengths and weaknesses of the monovariable chemical sensors and sensor arrays. It suggests that integrating multivariable sensing technology with carbon-based chemical sensors will further enhance their sensing performance in practical applications and enable the classification and identification of various analytes. The carbon-based multivariable chemical sensors focus on the efficient integration of sensing materials with transducers, optimizing the utilization of data collected by the sensing materials and the signals output by the transducers. This strategy fundamentally addresses the limitations inherent in monovariable sensors and sensor arrays. Since the inception of multivariable sensing [83], numerous classical and systematic reviews have emerged [8, 83–87]. This article focuses on multivariable chemical sensors composed of carbon-based materials and FETs, elucidating their fundamental principles and practical applications in multi-analyte recognition when integrated with pattern recognition algorithms. Among various multivariable extraction schemes, this paper provides an in-depth examination of techniques for extracting multiple output variables from the diverse output curves of FETs, a novel approach that continues to evolve alongside advancements in FET technology. It is hoped that the discussion of the latest technologies and application examples will further refine the theoretical framework of multivariable sensing and provide valuable theoretical foundations and technical references for the development of chemical sensor technologies. The organization of this article is as follows: Sect. 2 delves into the fundamental principles of carbon-based multivariable chemical sensors, encompassing carbon-based sensing materials, common sensor structures and fabrication processes, key performance metrics, pattern recognition algorithms, and multivariable sensing mechanism and feature extraction schemes. Section 3 explores various sensing technologies and application examples for extracting multivariable data from FET output curves for analyte identification.

## Carbon-Based Multivariable Chemical Sensors

Carbon-based multivariable chemical sensors, consisting of graphene/CNTs combined with multivariable transducers, have been applied to classify and recognize a wide range of analytes in complex chemical environments. This section will provide a concise overview of the following topics: 1) The development of carbon-based materials and their advantages in multivariable sensing. 2) Chemoresistive and five common FET-type transducers that are favorable for multivariable output. 3) Six performance metrics for quantitatively analyzing sensing performances. 4) Commonly used pattern recognition algorithms. 5) Two types of mechanisms for multivariable sensing. The objective is to present a comprehensive overview of the fundamental principles underlying carbon-based multivariable chemical sensors.

### Sensing Materials

#### Basic Properties of CNTs and Graphene

**Fig. 2:**
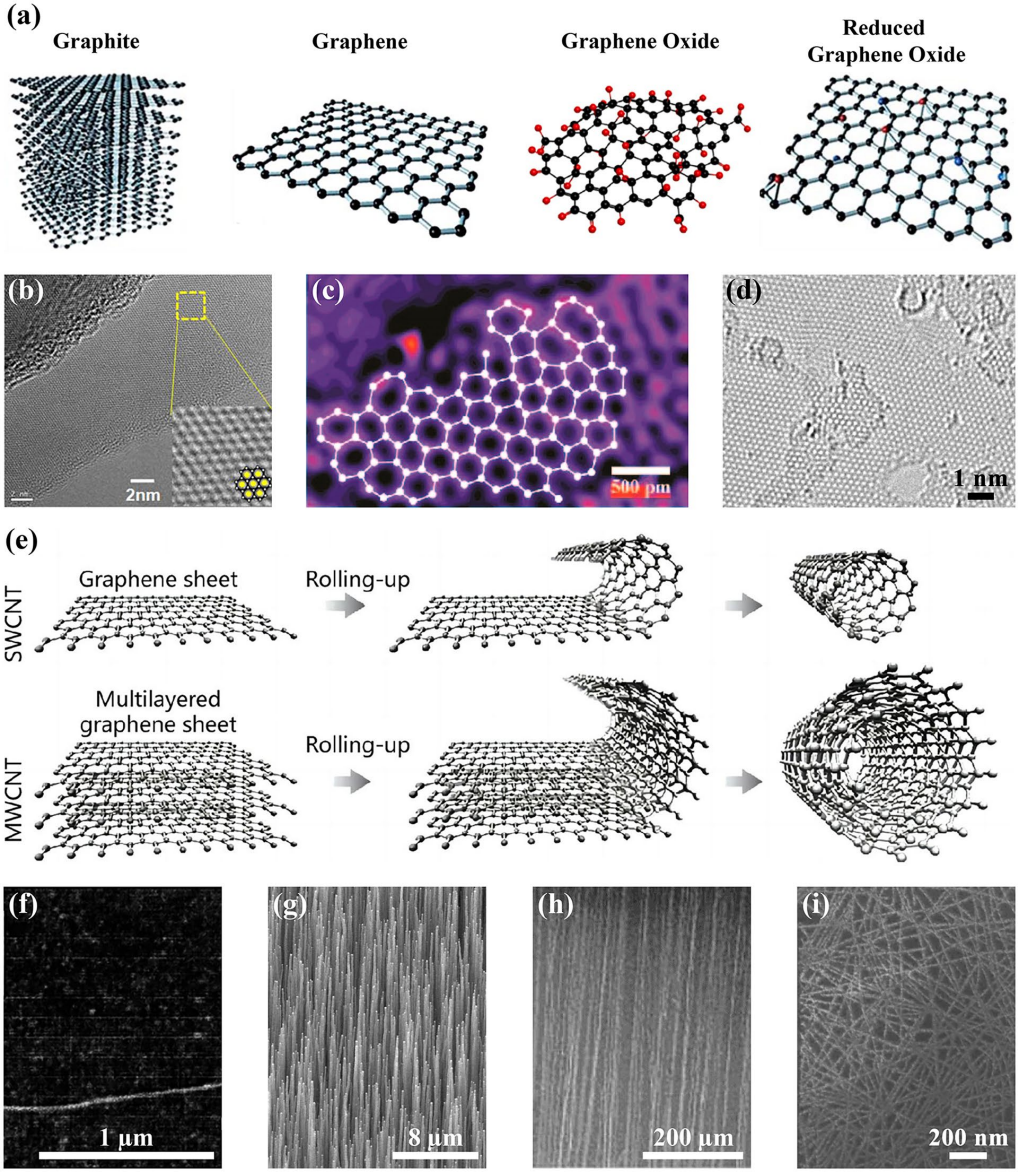
Structural schematic and microscopy diagrams of carbon-based materials. **a** Schematic of the three-dimensional structures of graphene, graphene oxide, and reduced graphene oxide isolated from graphite. Reproduced with permission [94]. Copyright 2024, Elsevier. **b** High-resolution transmission electron microscopy diagrams of graphene, **c** graphene oxide, and **d** reduced graphene oxide. **b** Reproduced with permission [89]. Copyright 2009, IOP Publishing. **c** Reproduced with permission [90]. Copyright 2016, American Chemical Society. **d** Reproduced with permission [91]. Copyright 2010, American Chemical Society. **e** Schematic diagram of single and multi-walled carbon nanotubes made of single and multi-layered graphene sheets rolled up. Reproduced under terms of the CC-BY license [104]. Copyright 2023, Royal Society of Chemistry. **f** Atomic force microscopy image of a single carbon nanotube. Reproduced with permission [111]. Copyright 2000, American Association for the Advancement of Science. **g** Scanning electron microscopy images of vertically aligned, **h** horizontally aligned, and **i** random networks carbon nanotubes. **g** Reproduced with permission [112]. Copyright 1998, American Association for the Advancement of Science. **h** Reproduced with permission [113]. Copyright 2006, American Chemical Society. **i** Reproduced with permission [114]. Copyright 2024, Elsevier

Carbon-based materials, in particular graphene and CNTs, are widely used for chemical sensing. For example, in 2004, Novoselov et al*.* pioneered a method for mechanically exfoliating graphene sheets from graphite using transparent adhesive tape, which initiated a surge of research activity in the field of graphene worldwide [88]. Figure 2a depicts the three-dimensional model diagrams of monolayer graphene, graphene oxide (GO), and reduced graphene oxide (rGO) isolated from graphite. The corresponding high-resolution transmission electron microscopy (HR-TEM) diagrams are presented in Fig. 2b–d, respectively [89–91]. Graphene and its derivatives are distributed as a honeycomb network in a two-dimensional plane, which gives them a specific surface area of up to 2630 m^2^ g^−1^ [92], a carrier mobility of about 1400 cm^2^ V^−1^ s^−1^ [88], and an excellent thermal conductivity of up to about 50,000 W mK^−1^ [93]. The oxidation of graphite results in the introduction of several oxygen-containing functional groups (CO, OH, and COOH) on its surface, leading to the formation of GO with a larger surface area and good hydrophilicity [94, 95]. The reduction of GO yields rGO with less than 10% oxygen content [96], which exhibits properties intermediate between those of GO and graphene [97]. This derivative is therefore a more suitable choice for large-scale fabrication [98]. Graphene and its derivatives can be readily functionalized to enhance the selectivity and sensitivity, rendering it an optimal material for the development of chemical sensors [99, 100]. In 2007, Schedin et al*.* pioneered the use of graphene in gas sensing by employing graphene-based FET for the initial detection of NO_2_ [101].

In 1991, Iijima observed the appearance of needles near fullerenes using HR-TEM and designated them as carbon nanotubes, a discovery that is regarded as pivotal in the field of CNTs research [102]. As illustrated in Fig. 2e, CNTs can be conceptualized as one-dimensional hollow cylinders constituted by graphene sheets rolled along a chiral vector [103, 104], wherein the carbon atoms are predominantly characterized by *sp*^2^ hybridization, and the curved hexagonal lattice also gives rise to *sp*^3^ hybridization bonds [105]. This distinctive structure endows them with robust electrical and chemical properties, high mechanical strength, and favorable thermal stability [106, 107]. The distinctive one-dimensional structure and hanging-bond-free surface of CNTs diminish the scattering probability, thereby enhancing the carrier mobility (~ 100,000 cm^2^ V^−1^ s^−1^) and mean free range (~ 1 µm) [108], which can markedly accelerate the speed of carbon-based electronic devices. The categorization of CNTs is based on two main criteria: the number of graphene sheet layers and the chirality. Single-walled and multi-walled CNTs are distinguished by the number of graphene sheet layers [109], while semiconducting and metallic CNTs are categorized based on their chirality [110]. The morphology of CNTs on the substrate allows for their categorization into single CNT and CNTs networks (Fig. 2f–i) [111]. The latter can be further categorized into vertically aligned (Fig. 2g) [112], horizontally aligned (Fig. 2h) [113], and random networks (Fig. 2i) [114]. In 2000, Kong et al. conducted the inaugural study of NO_2_ and NH_3_ sensing using a FET-type gas sensor comprising a single-walled CNT. This pioneering work paved the way for a new avenue of research into carbon-based gas sensors [111].

#### Fabrication and Applications of Wafer-Scale Carbon-Based Devices

It is evident from the success of silicon-based electronic devices that transitioning from primitive carbon-based materials to carbon-based sensors for practical applications necessitates the scalable, wafer-level fabrication of carbon-based devices. Liyanage et al. employed a semiconducting CNTs solution, prepared via regioregular poly(3- dodecylthiophene)-assisted sorting, to fabricate wafer-scale devices featuring CNTs random networks [115]. Using a cost-effective solution processing method, the resulting carbon-based devices exhibited an average mobility of 1 cm^2^ V^−1^ s^−1^, a *I*_on_/*I*_off_ of 10^6^, and a 100% yield, presenting a viable pathway for wafer-scale carbon-based device fabrication. Similarly, to achieve large-area single-walled CNTs films on transparent plastic substrates, Kiriya et al. employed solution processing combined with a roll-to-roll (R2R) approach, achieving CNTs film coverage of 99% [116]. This study has paved the way for the fabrication of large-area, flexible carbon-based sensor devices. Distinct from the commonly used solution processing methods for CNTs wafers, Yuan et al. demonstrated a stacking transfer technique to transfer chemical vapor deposition (CVD)-grown graphene from flat to flat, enabling the fabrication of wafer-scale graphene-based van der Waals superlattices with controlled twist angles [117]. This work laid a solid foundation for the development of wafer-scale graphene-based sensors and provided ample opportunities for further advancements. Jiang et al. reviewed the controlled synthesis of wafer-scale graphene films using CVD [118]. In their review, the authors emphasized the critical roles of chemical kinetics and fluid dynamics in enabling the scalable production of graphene films, while discussing the industrial prospects and potential directions for wafer-scale graphene film synthesis. Excitingly, Bishop et al. reported the fabrication of carbon nanotube field-effect transistors (CNFETs) on industry-standard 200 mm wafers using equipment compatible with existing silicon-based electronic device fabrication processes [119]. In their work, the authors evaluated the electrical performance of 4,800 individual CNFETs on a single wafer, finding that all key performance metrics exhibited narrow distributions with minimal spatial dependence across the wafer, thus demonstrating the consistency of wafer-scale device performance. To validate the repeatability and reliability of the fabrication process, the authors conducted repeated tests on three wafers from the same batch, achieving a 100% device yield (14,400/14,400). The demonstrated process, which is highly compatible with silicon CMOS technology, exhibited excellent uniformity and reproducibility, paving the way for the commercialization of carbon-based devices.

Benefiting from mature carbon-based wafer fabrication techniques, numerous studies on sensor devices fabricated on carbon-based wafers have demonstrated excellent consistency and reliability [56, 120–125]. For instance, Liang et al. developed wafer-scale uniform CNT biosensors capable of specifically detecting DNA and microbubbles, which are biomarkers of diseases [122]. Testing of 90 randomly selected liquid-gated CNTFETs on the wafer revealed highly consistent transfer characteristic curves, providing a highly reliable platform for the development of high-sensitivity carbon-based biosensors. Soikkeli et al. utilized standard commercial techniques to fabricate wafer-scale graphene field-effect transistor (GFET) biosensor arrays with CMOS readout circuits on 200 mm wafers [125]. Their work demonstrated an impressive device yield of 99.9% (2,558/2,560), with an average sensitivity of 42 mV decade^−1^ (SD = 4 mV decade^−1^) for 512 GFETs in response to NaCl concentrations ranging from 1 to 100 mM. The biosensor arrays exhibited excellent uniformity and reproducibility, while the integration of CMOS multiplexing circuits for multi-analyte sensing and statistical analysis significantly simplified the complexity of practical biosensing applications. The advancements in carbon-based wafers and wafer-scale chemical sensors highlight the robust device foundation for constructing carbon-based multivariable sensors. This can be attributed to the stable chemical properties and reliable physical parameters of carbon-based materials, enabling seamless compatibility with popular silicon CMOS processes.

Compared to graphene-based materials, CNTs intended for use in carbon-based chemical sensors must exhibit high semiconducting purity. However, CNTs prepared via mainstream methods such as solution filtration or CVD typically consist of approximately 67% semiconducting CNTs and 33% metallic CNTs [126]. The presence of even a small amount of metallic CNTs significantly increases the electrical conductivity of CNTs film, thereby compromising their semiconducting properties, which is detrimental for sensing applications. In laboratory settings, conjugated polymer-based purification techniques have enabled the preparation of CNTs with semiconducting purity as high as 99.9999% [127]. Currently, commercial semiconducting CNTs, such as IsoNanotubes-S from NanoIntegris, achieve purities up to 99.9%, but their cost remains high. To advance the commercialization and industrialization of carbon-based materials, it is imperative to reduce the production costs of high-performance materials at the source. Only then can these materials be effectively integrated with mature silicon-based CMOS processes, paving the way for widespread adoption in everyday applications.

#### Carbon-Based Materials: An Ideal Choice for Multivariable Sensing

In multivariable chemical sensors, the transducers are capable of outputting multiple electrical parameters that reflect the physicochemical property changes of sensing materials, thereby enabling the classification and identification of multiple analytes with only one sensing material [8, 83–87]. This represents a fundamental difference from conventional sensor arrays, which require sensing units modified with different sensing materials. Weimar and Göpel were the first to systematically discuss the concept and development trends of multivariable chemical sensors (then referred to as multiparameter sensor systems) [83]. In their work, the authors described a chemical sensor composed of a single sensing material combined with multi-transducers. In earlier research, Weimar et al. simultaneously measured changes in work function, conductance (G), and catalytic activity of a single Figaro gas sensor in the presence of different analytes using various characterization techniques [128]. The experimental results demonstrated that a single Figaro sensor could determine the partial pressures of CO and H_2_O in air, successfully proving that a single sensing material could produce differentiated responses to different analytes, laying the foundation for the development of subsequent multivariable sensors. Huang and Hayward designed an orthogonal ambipolar semiconductor composed of a p-type polymer and vertically in-plane aligned n-type small-molecule nanowires. Using a single FET fabricated with this sensing material, they successfully discriminated 22 different volatile organic compounds [129]. The orthogonal charge-carrying pathways and p-n junctions provided by this sensing material not only enabled responses to a wide range of analytes but also allowed discrimination of closely related derivatives with single-atom resolution. This work leveraged the differential response capability of a single sensing material, combining it with eight output variables extracted from the transfer characteristic curves to achieve multi-analyte recognition.

In recent studies of carbon-based sensors, Shi et al. achieved the identification of six gases (NO_2_, NH_3_, H_2_, H_2_S, CO, and SO_2_) using only Pd nanoparticle-modified CNTs [130]. Hayasaka et al. utilized a single graphene-based FET for the recognition of water, methanol, and ethanol [131]. Similarly, Agbonlahor et al. employed a single graphene-based FET to identify four analytes in the environment [132]. These studies have successfully validated that carbon-based materials can also generate differentiated responses to various analytes, making them ideal candidates for sensing materials in multivariable chemical sensors.

In this section, a concise overview of the sensing materials constituting carbon-based multivariable chemical sensors is provided, encompassing their fundamental properties, wafer-level fabrication and applications, as well as the distinctive characteristics of their differentiated responses. The selection of CNTs/graphene as the ideal candidates for multivariable sensors can be attributed to the following three key aspects: 1) The highly compatible preparation flow with CMOS process enables batch preparation at the wafer level [122, 127]. 2) Low power consumption and room temperature operating characteristics make these sensors eminantly suitable for use in portable devices [133, 134]. 3) The large specific surface area resulting from the low-dimensional structure rendering it highly sensitive to the immediate chemical environment, thereby enabling single-molecule sensitivity [124, 135]. The differing amounts of charge transfer and intensities of scattering to carriers, which are caused by the adsorption of analytes with different chemical properties, are the fundamental source of the differentiated response [101, 136].

### Sensor Structures and Fabrication Processes

#### Sensor Structures

**Fig. 3:**
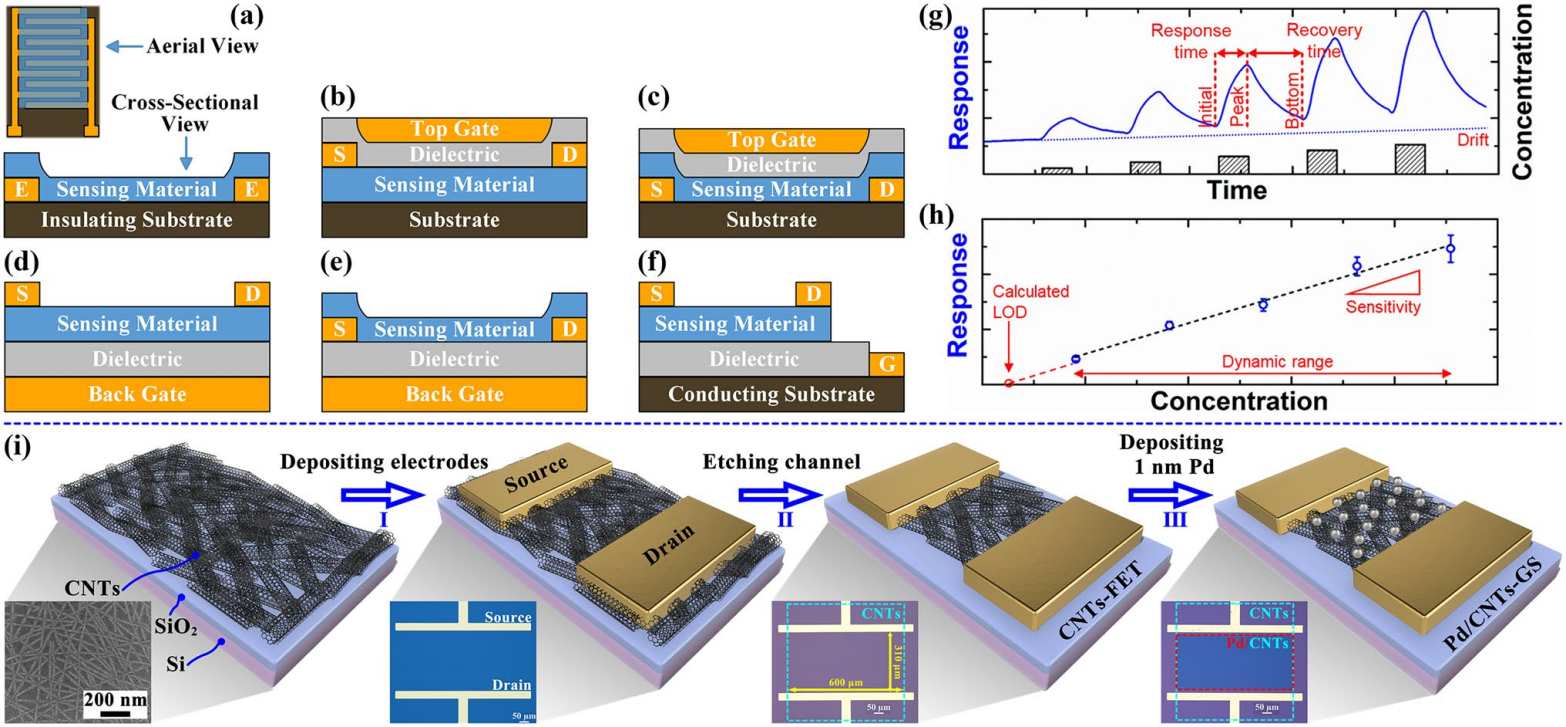
Schematic structures of **a** chemoresistive and five field-effect transistor-type chemical sensors for: **b** top-gate top-contact, **c** top-gate bottom-contact, **d** back-gate top-contact, **e** back-gate back-contact, and **f** side-gate devices. Performance metrics reflecting the sensing performance in **g** response curve and **h** calibration curve. Reproduced with permission [72]. Copyright 2019, American Chemical Society.** i** Processes for preparing carbon-based field-effect transistor-type chemical sensors. Reproduced with permission [130]. Copyright 2024, American Chemical Society

Multivariable chemical sensors have a variety of possible device structures, however due to space limitations we will focus on chemoresistive and FET semiconductor-type chemical sensors. Figure 3a shows the aerial and the cross-sectional view of a typical chemoresistive device. Chemoresistive sensors are two-terminal devices consisting of an interdigital electrode and sensing material on the insulating substrate. The interaction of the analyte with the sensing material alters the resistance between the interdigital electrodes. The multivariable outputs are typically extracted using response-time curves.

The FET-type chemical sensors are three-terminal devices comprising a source, drain, and gate. The source and drain are in direct contact with the sensing material, while the gate is isolated from the sensing material by a dielectric layer. During operation, the channel carrier concentration is controlled by applying the gate-source voltage (*V*_gs_) to achieve an optimal sensor sensitivity. Concurrently, the drain-source voltage (*V*_ds_) is applied to inject carriers from the source into the sensing material, which subsequently transfers to the drain. The categorization of FET-type chemical sensors is dependent upon the gate position. The classification of these sensors includes top-gate [137], back-gate [138], side-gate [139], suspended-gate [140], and horizontal floating-gate [141]. The top-gate (Fig. 3b, c), back-gate (Fig. 3d, e), and side-gate (Fig. 3f) devices are the most commonly used designs in multivariable sensing applications. Depending on the position of the source-drain electrodes relative to the sensing material, the top gate can be categorized into the top-gate top-contact type (TGTC, Fig. 3b) and the top-gate bottom-contact type (TGBC, Fig. 3c), and the back gate can be categorized into the back-gate top-contact type (BGTC, Fig. 3d) and the back-gate back-contact type (BGBC, Fig. 3e). The TGTC and TGBC devices demonstrate excellent long-term stability due to the gate and the dielectric layer covering the surface of the sensing material, which provides robust protection. The analyte’s inability to directly contact the sensing material results in a low response. To address this challenge, metals with a catalytic effect can be employed as the gate material, and the dipole layer generated by the reaction with the analyte can be leveraged for channel carrier concentration regulation [142]. The sensing materials of BGTC devices are prepared directly on the dielectric surface with a simpler interfacial environment, which allows for enhanced gate control and superior sensor performance [143]. However, it is important to consider the impact of the subsequent source-drain electrodes deposition process on the sensing material, as well as the compatibility with the CMOS processes [144]. In contrast, BGBC devices have better process compatibility and are ideal for batch preparation. Nevertheless, their high contact resistance and irregular sensing material surfaces can affect carrier transport [143]. The transition from laboratory product to commercialized product necessitates the use of sensors with optimal packability. As shown in Fig. 3f, the electrode position distribution of side-gate devices is not only compatible with the prevailing lead bonding processes but also ensures the complete exposure of the sensing material to the analytes [139]. Notwithstanding, the use of small, non-vertically opposed gates necessitates the application of larger *V*_gs_ for channel modulation.

#### Fabrication Processes

Figure 3i illustrates an example of the fabrication processes for the BGTC carbon-based chemical sensor [130], including device schematic diagrams, scanning electron microscope (SEM) image, and optical photographs for each step. The entire fabrication process consists of three steps and is compatible with silicon-based CMOS technology. In the first step, the semiconducting single-walled CNTs film with a random network morphology is deposited on a silicon substrate using an immersion deposition method. Step I involves defining the source/drain electrode regions on the carbon-based substrate using photolithography, followed by electron-beam evaporation to deposit the source/drain electrodes (Ti/Pd/Au, 0.6/20/40 nm). It is noteworthy that the choice of source and drain electrode materials determines whether the fabricated FET device is p-type or n-type. High work function metals such as Pt and Pd are used to form p-type ohmic contacts with CNTs [145, 146], while low work function metals such as Sc are used to form n-type ohmic contacts with CNTs [147]. Ti, which exhibits good wettability with CNTs, is employed as an adhesion layer between the source/drain electrodes and the CNTs [148]. Pd, as a high work function metal, forms Schottky contacts with CNTs that are barrier-free for hole transport [149]. Au is added to increase the electrode thickness, facilitating performance testing. Step II begins with defining the channel region between the source and drain electrodes via photolithography. The CNTs film outside the channel region is then removed using reactive ion etching, resulting in a carbon-based FET (CNT-FET) with a back gate structure. To enhance sensing performance, the CNTs in the channel region can be functionalized. In Step III, the 1 nm Pd nanoparticles are deposited on the channel region via electron-beam evaporation to enhance the catalytic capability of the sensor. The fabrication process of the BGTC carbon-based sensor is straightforward and, when combined with the mature carbon-based wafer preparation techniques and industrial silicon-based CMOS processes, has the potential to accelerate its commercialization.

### Key Performance Metrics

In order to quantitatively analyze the sensing performances of chemical sensors, performances are typically evaluated using key performance metrics such as response/recovery time, drift, sensitivity, limit of detection (LoD), selectivity, and long-term stability. Typical response curve and calibration curve, shown in Fig. 3g, h, help to graphically understand the specifics of what is quantified by these performance metrics [72]. The raw sensing data obtained typically consists of response curves that track changes over time under varying analyte concentrations. The response is commonly defined using three mainstream approaches: ΔX/X₀, X/X₀, or simply ΔX, where X can represent resistance (R), current (I), capacitance (C), inductance (L), or conductance (G). In the context of multivariable sensing applications based on FETs, X can be further extended to parameters such as threshold voltage (*V*_th_), transconductance (*g*_m_), or subthreshold swing (*SS*), etc. which reflect changes in the electrical characteristics of the FETs.

As shown in Fig. 3g, the term response time is defined as the time elapsed between the initial exposure of the sensor to the analyte and the point at which the response reaches 90% of its peak value [150]. The calculation formula can be defined as shown in Eq. (1):

1 $$\begin{array}{*{20}c} {{\text{Response}}\;{\text{time}}\;{ = }\;0.9\left( {{\text{Time}}_{{{\text{peak}}}} - {\text{Time}}_{{{\text{initial}}}} } \right)} \\ \end{array}$$

where, ${Time}_{peak}$ refers to the time at which the response reaches its peak, and ${Time}_{initial}$ represents the time when the sensor first comes into contact with the analyte. In contrast, the term recovery time is defined as the time required for the sensor to return to its original state following the removal of the analyte, until the response value drops to 10% of its peak value [72]. The calculation formula can be defined as shown in Eq. (2):

2 $$\begin{array}{*{20}c} {{\text{Recovery}}\;{\text{ time}}\; = \;0.9\left( {{\text{Time}}_{{{\text{bottom}}}} - {\text{Time}}_{{{\text{peak}}}} } \right)} \\ \end{array}$$

where, ${Time}_{bottom}$ refers to the time at which the response recovers to its lowest value. The recovery time is typically regarded as the inverse of the response time, with a rapid response time often accompanied by a slower recovery time due to chemisorption [151].

The term drift is defined as the gradual, non-random alteration in the sensor’s response over time when the sensor is exposed to a constant analyte concentration or a blank sample [152]. Effective passivation or drift elimination algorithms can be utilized to mitigate the effects of drift [153]. The drift rate over a testing period $t$ can be defined using the formula shown in Eq. (3):

3 $$\begin{array}{*{20}c} {{\text{Drift}}\;{\text{ratio}}\;{ = }\;\frac{{R_{t} - R_{0} }}{{R_{0} }} \cdot \frac{1}{{t - t_{0} }} \cdot 100\% \left( {\% /h} \right)} \\ \end{array}$$

where, ${R}_{t}$ and ${R}_{0}$ represent the response value at time $t$ and the initial response value, respectively. Albarghouthi et al. measured the open-circuit potential drift rate of CNT-TFTs over a one-hour period, quantified as the first derivative of the open-circuit potential, which was approximately 14 mV h^−1^ [154]. This approach was employed to investigate the charge screening and drift limitations of BioFETs, providing valuable insights that could enhance the sensitivity and robustness of the sensors.

According to the International Union of Pure and Applied Chemistry (IUPAC) [155], sensitivity is defined as the ability to provide a reliable and measurable response to changes in analyte concentration. As shown in Fig. 3h, the calculation formula can be defined as Eq. (4) [156]:

4 $$\begin{array}{*{20}c} {{\text{Sensitivity}}\; = \;{\text{slope}}\;{\text{of}}\;{\text{calibration}}\;{\text{curve}}\; = \;\frac{{{\text{change}}\;{\text{in}}\;{\text{response}}}}{{{\text{change}}\;{\text{in}}\;{\text{analyte}}\;{\text{concentration}}}}} \\ \end{array}$$

Sensitivity is the most intuitive parameter for characterizing the sensor performance. A high-performance sensor can produce a linear response over a wide analyte concentration range (dynamic range), which corresponds to a constant sensitivity [157]. This greatly simplifies the design complexity of backend pattern recognition algorithms and readout circuits.

The LoD is defined as the lowest concentration that can be reliably detected as not present in the sample at a 99% confidence interval [158]. Figure 3h shows the calculated LoD, which can be defined using the formulas in Eqs. (5) and (6) [155]:

5 $$\begin{array}{*{20}c} {R_{\min } \; = \;\overline{R}_{b} + ks_{b} } \\ \end{array}$$

6 $$\begin{array}{*{20}c} {LoD\; = \;\frac{{R_{\min } - \overline{R}_{b} }}{{{\text{sensitivity}}}}} \\ \end{array}$$

where, ${\overline{R} }_{b}$ and ${s}_{b}$ represent the mean response value and the standard deviation obtained from $n$ measurements of the blank sample, respectively. $k$ is a numerical factor corresponding to the selected confidence level, also referred to as the confidence factor. IUPAC recommends that $n$ should exceed 20, and for a confidence level of 99.6%, $k$ is set to 3. It is notable that the LoD could also be influenced by the dissociation constant of the analyte in biosensors [159]. As can be seen from the calculation formulas, LoD is closely related to the sensitivity of the sensor and the noise level. Enhancing the interaction strength between the sensing material and the analyte improves sensitivity [160], while reducing the intrinsic noise of the sensing material and the transducer increases the signal-to-noise ratio (SNR) [161], effectively lowering the LoD.

Selectivity is defined as the ratio of a sensor’s response to a target analyte to its response to an interfering analyte [153]. The calculation formula can be defined as shown in Eq. (7) [156]:

7 $$\begin{array}{*{20}c} {{\text{Selectivity}}\;{\text{coefficient}}\; = \;K_{A, X} \; = \;\frac{{{\text{response}}\;{\text{to}}\;X}}{{{\text{response}}\;{\text{to}}\;A}}} \\ \end{array}$$

where, $X$ represents the interfering analyte, while $A$ denotes the target analyte. A smaller Selectivity coefficient indicates that the sensor is more resistant to the influence of $X$, thus exhibiting better selectivity. For multivariable sensors, the sensing materials used are required to exhibit differential responses to various analytes. Quantitatively, this means having different selectivity coefficients. The use of multivariable sensors can be an effective means of improving selectivity in complex chemical environments.

Long-term stability is defined as the ratio of the aged sensor response to that of a newly prepared sensor, and is employed to assess the capacity of a sensor to generate consistent output for a given input over time [162]. Previous studies did not provide a formalized definition of long-term stability. We have proposed a formula for evaluating device stability based on the drift rate. Similar to Eq. (3), the calculation formula for long-term stability over a testing period $t$ can be defined as shown in Eq. (8):

8 $$\begin{array}{*{20}c} {{\text{Stability}}\; = \;\frac{{R_{t} - R_{0} }}{{R_{0} }} \cdot \frac{1}{{t - t_{0} }} \cdot 100\% \left( {\% /{\text{day}}} \right)} \\ \end{array}$$

where, $t$ is typically set to 15 days, 30 days, 60 days, six months, or one year. Zhao et al. investigated the long-term stability of the MX-s@NiMo-P sensor by monitoring its response to 100 ppm NH_3_ over a 30-day period. The sensor’s response decreased from 188.91% on the first day to 176.48% on the 30th day. This analysis provides valuable insights into the sensor’s degradation behavior and failure mechanisms [163].

### Pattern Recognition Algorithms

In practical applications, the selection of suitable and efficient pattern recognition algorithms plays a critical role in fully leveraging the advantages of multivariable sensing, thereby enhancing classification and recognition performance. Numerous studies have systematically reviewed various pattern recognition algorithms [164–171]. This section provides a concise summary of commonly used algorithms, with the key characteristics and comparisons presented in Table 1. Pattern recognition algorithms widely used for analyte classification and identification include classical algorithms such as Principal Component Analysis (PCA), Linear Discriminant Analysis (LDA), Support Vector Machine (SVM), and k-Nearest Neighbor (KNN), as well as neural network-based algorithms like Feedforward Neural Networks (FNN), Recurrent Neural Networks (RNN), and Convolutional Neural Networks (CNN). While each algorithm has specific application scenarios, the general workflow of pattern recognition typically involves the following steps [165]: 1) Data Acquisition and Preprocessing: Collect raw data from chemical sensors or similar devices, which are often multi-dimensional signal responses. Preprocessing is performed to enhance classification accuracy and algorithm robustness [167]. 2) Feature Extraction: Extract key features from the raw data, such as maximum response, response time, or curve gradients, to reduce redundant information. 3) Dimensionality Reduction: Apply algorithms such as PCA to reduce data dimensionality while retaining critical information, facilitating subsequent classification modeling. 4) Classification Modeling and Training: Use classification algorithms to construct models that learn the relationship between input features and class labels based on training data. 5) Testing and Validation: Input test data into the model to evaluate its classification accuracy and robustness.

**Table 1:** Summary and comparison of common pattern recognition algorithms

Algorithm		Category	Advantages	Disadvantages
Classical algorithm	PCA	Unsupervised	Fast and efficient, suitable for high-dimensional data	Applicable only to linear data
		(Classification/Dimensionality reduction)	Easy to visualize	May lose nonlinear features
	LDA	Supervised	Simple computation	Susceptible to outliers
		(Classification/Dimensionality reduction)	Performs well on linearly separable data	Restrictions on the feature dimension
	SVM	Supervised	Perform well on high-dimensional, small-sample data	Inefficient for large datasets
		(Classification/Regression)	Support nonlinear classification	Require parameter tuning and is sensitive to kernel function selection
	KNN	Supervised	Simple and intuitive	Inefficient for high-dimensional data
		(Classification/Regression)	No training required	Susceptible to the value of K
				Susceptible to outliers
Neural network algorithm	FNN	Supervised&Deep learning	Simple and intuitive	Susceptible to overfitting
		(Classification/Regression)	Have strong universality	Unsuitable for dealing with time series data
	RNN	Supervised&Deep learning	The capacity to handle sequential data	Poor at parallel computing
		(Classification/Regression)	Grasp the contextual information	High computational cost
	CNN	Supervised&Deep learning	Automatically extracts features	High computational resource requirements and training costs
		(Classification/Regression/Feature extraction)	Parameter sharing leads to efficient computation	Heavily reliant on large amounts of data

PCA is an unsupervised algorithm commonly used for dimensionality reduction and feature extraction. PCA reduces the dimensionality of data by computing the eigenvalues and eigenvectors of the covariance matrix of the dataset [172]. The eigenvectors corresponding to the largest eigenvalues are selected as the principal components. The data is then projected onto these principal components, achieving dimensionality reduction. It reduces data dimensionality while retaining the maximum variance, providing critical support for subsequent classification tasks [173]. LDA, a supervised algorithm, maximizes the ratio of between-class variance to within-class variance based on data categories, generating optimal classification boundaries and proving effective for multi-class classification tasks [174]. The core computational principle of LDA is to solve the generalized eigenvalue problem that maximizes the ratio of the between-class scatter matrix to the within-class scatter matrix [175]. This yields the optimal projection directions. By projecting the data onto these directions, LDA achieves dimensionality reduction and classification. SVM is particularly suited for high-dimensional and non-linear problems, constructing optimal hyperplanes to separate different classes, and demonstrating excellent generalization performance [176]. The core computational principle of SVM is to find an optimal hyperplane in the feature space that maximizes the margin between two classes of samples. For linearly inseparable problems, SVM uses a kernel function to map the data into a higher-dimensional space where it becomes linearly separable, and then determines the optimal hyperplane in that space [177]. KNN, a simple distance-based classification algorithm, determines class membership by comparing the similarity between the test samples and training samples. It is effective for small-scale datasets but has high computational complexity [178]. The core computational principle of KNN is to classify a given test sample by calculating its distances to all samples in the training set, selecting the K nearest neighbors, and inferring the class of the test sample based on the classes of these neighbors [179]. Typically, the final class is determined using majority voting or similar strategies among the K neighbors.

In practical applications, a large amount of nonlinear sensor data exists. Neural network algorithms, which mimic the way the biological brain processes complex problems, are designed to perform tasks such as data classification, regression, and prediction. These algorithms are considered powerful tools for handling nonlinear sensor data [171]. FNN, as the most basic type of neural network, consists of an input layer, hidden layers, and an output layer. Information flows only in one direction, from the input layer to the output layer, without feedback loops. The core operating principle involves passing input data through the input layer, processing it in the hidden layers by performing weighted summation of input signals at neurons, followed by nonlinear transformations via activation functions, and finally outputting results at the output layer [180]. This algorithm’s simple structure and computational efficiency make it suitable for most classification and regression tasks [181]. A multilayer FNN with backpropagation learning capability is referred to as a Backpropagation Neural Network (BPNN), which adjusts weights based on errors to enable learning. This allows it to handle more complex nonlinear problems [182]. To enhance the ability of FNN to process time-series data, RNN with cyclic connection structures were developed. In RNN, the hidden units include a state vector that retains historical information from past elements in the sequence [183]. Information is cyclically passed through the neurons, allowing historical information to influence the current output, thereby enabling the network to learn temporal dependencies in sequential data. This characteristic makes RNNs particularly well-suited for handling time-series data, such as the analyte concentration time series measured by chemical sensors, effectively capturing the dynamic features in response-time curves [184, 185]. Unlike the previously mentioned neural network algorithms, CNN consists of convolutional layer, activation function, pooling layer, fully connected layer, and output layer. The core operating principle of CNN is to extract local features by performing convolution operations using kernels that slide over the data in the convolutional layers. The pooling layers are then used to compress and reduce the dimensionality of the features, and the fully connected layers integrate these extracted features to produce the final output [186, 187]. The presence of convolutional and pooling layers enables CNNs to automatically extract critical features from raw data, making them more efficient than fully connected networks when handling high-dimensional data [188].

### Multivariable Sensing Mechanism and Feature Extraction Schemes

Since the pioneering concept of multivariable sensing was first introduced by Weimar and Göpel [83], this field has undergone substantial development both theoretically and practically. The initial framework was significantly advanced by Hierlemann and Gutierrez-Osuna, who expanded the concept to encompass higher-order sensing paradigms [85]. Subsequently, Potyrailo provided a comprehensive systematic review of various multivariable sensors in the context of the IoT and industrial internet applications [8]. This progressive evolution has established multivariable sensing as a mature and versatile technology with extensive practical implementations. In this section, we will focus on analyzing the working mechanism of carbon-based multivariable sensors and present two main ways of extracting multiple output variables.

#### Multivariable Sensing Mechanism

**Fig. 4:**
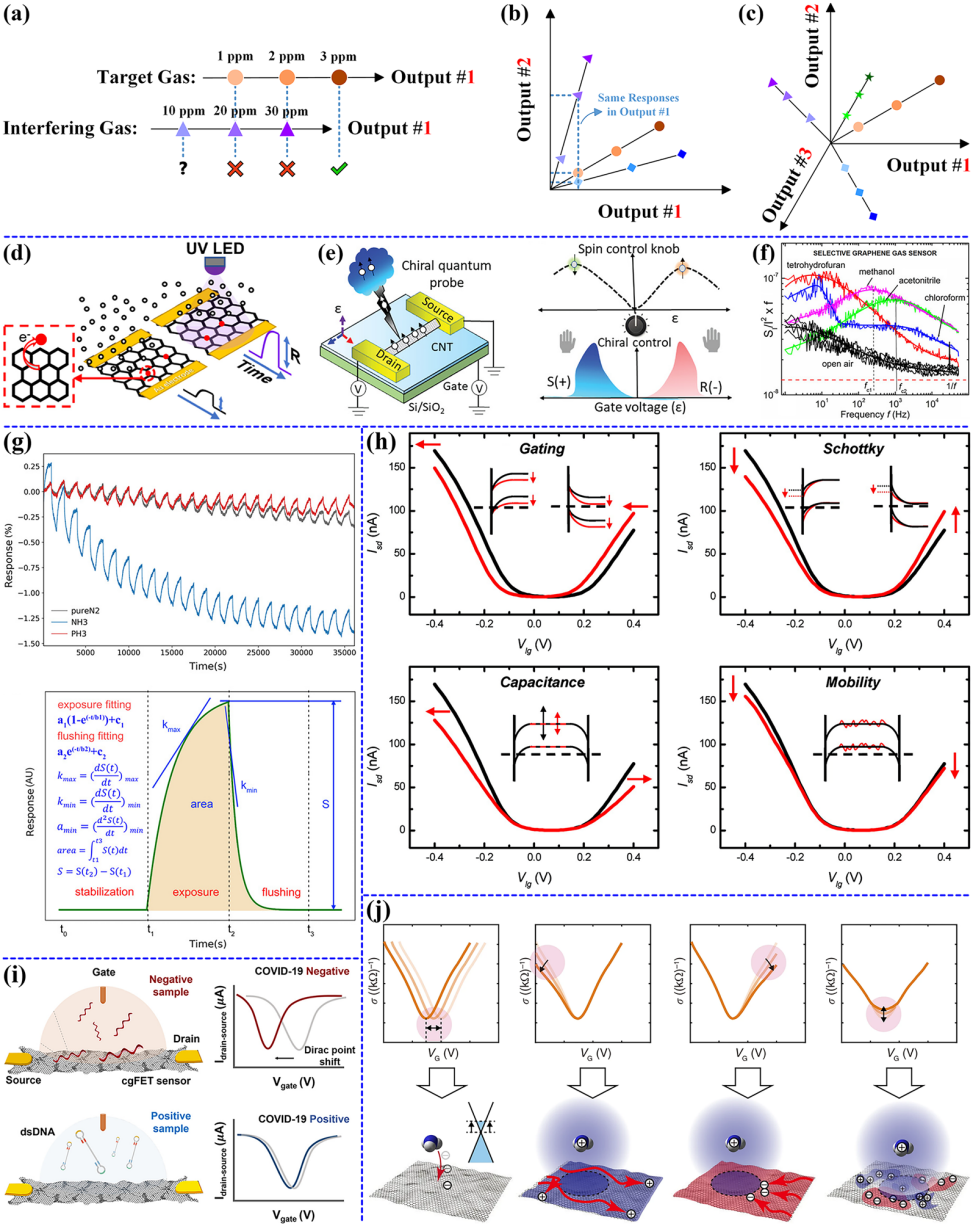
**a** Overlap in the responses observed between the interfering and target gas when only a single output variable (Output #1) is utilized. Sensor response to interfering gases: 10 ppm caused anomalous output (?); 20–30 ppm responses overlapped with 1–2 ppm target gas ( ×); only 3 ppm target gas overcame interference (√). **b** Output characteristic curves for three analytes in a two-dimensional sensing plane with two output variables (Output #1 and Output #2). While all three analytes show identical responses in Output #1, they display distinct patterns in Output #2, enabling successful differentiation. **c** Output characteristic curves of four analytes in a three-dimensional sensing space with three output variables (Output #1, Output #2, and Output #3). Higher-dimensional sensing space demonstrates enhanced capability for analyte discrimination, enabling identification of a broader range of chemical species. **d** Output variables extraction using light modulation and **e** gate voltage modulation. **d** Reproduced with permission [54]. Copyright 2021, American Chemical Society. **e** Reproduced with permission [211]. Copyright 2023, Wiley–VCH. Extraction of output variables from **f** noise spectra and **g** response-time curves. **f** Reproduced with permission [215]. Copyright 2012, American Chemical Society. **g** Reproduced under terms of the CC-BY license [217]. Copyright 2022, Wiley–VCH. **h** Four variation patterns of transfer characteristic curves of carbon nanotube-based FET-type chemical sensors capable of reflecting the sensing mechanisms. Reproduced with permission [218]. Copyright 2008, American Chemical Society. **i** Schematic illustration of COVID-19 positive and negative samples differentiation using the Dirac point offset of graphene-based FET-type chemical sensors. Reproduced with permission [225]. Copyright 2021, American Chemical Society. **j** Four variation patterns of transfer characteristic curves of graphene-based FET-type chemical sensors that reflect the sensing mechanisms. Reproduced under terms of the CC-BY license [131]. Copyright 2020, Nature Publishing Group

Figure 4a illustrates the output characteristic curves of a monovariable sensor for a target gas of 1–3 ppm and an interfering gas of 10–30 ppm. The output parameter, designated Output #1, can be resistance, voltage, current, or work function, contingent on the specific sensor type. When only the target analyte is present in the environment, or when the concentration of interfering analytes is low, Output #1 can easily be relied upon for quantitative analysis of analyte concentrations. In the event that the concentrations of interfering analytes are too high, it becomes challenging to accurately detect the target analyte by relying on Output #1 alone. For instance, a 10 ppm interfering gas produces an unintended output, and outputs produced by 20–30 ppm interfering gas cause misjudgment, leading to a false sense of successful detection of the 1–2 ppm target gas. Several specific designs have been proposed to enhance the selectivity of monovariable sensors in complex environments [189–191]. For example, van den Broek et al. employed a Tenax TA separation column to isolate methanol from interfering substances such as ethanol, acetone, or hydrogen, thereby achieving selective detection of formaldehyde [191]. This sensor demonstrated the ability to detect methanol in the range of 1 to 1,000 ppm even in the presence of ethanol concentrations as high as 62,000 ppm. Similar to a gas chromatography column, the nonpolar adsorbent Tenax TA separates analytes in a mixture based on molecular weight and functional groups. This ensures that target analytes and interfering substances reach the sensor at different times, thereby avoiding overlapping responses. Using the same technique, Abegg et al. and Cao et al. achieved highly selective detection of methanol and benzene, respectively [189, 190]. In addition, the construction of various composite materials, such as polymer/CNTs [192], Fe/CNTs [193], and MOF/CNTs [194], has also proven effective in improving sensor selectivity.

Through these studies, the selectivity of monovariable sensors can be significantly enhanced, enabling them to overcome the interference from coexisting analytes and thereby achieve the detection of a single analyte in complex chemical environments. However, when addressing the task of simultaneous detection of multiple analytes, monovariable sensors modified with either specific or cross-sensitive materials demonstrate limitations. Specifically, the former fails to generate differentiated response signals for distinct analytes, while the latter relies solely on a single output variable. Although the construction of sensor arrays composed of multiple specifically modified monovariable sensors can facilitate multi-analyte detection through a "one-key-one-lock" approach, this method necessitates sophisticated modification of multiple sensing units according to the target analytes [195, 196]. Moreover, with the increasing variety of analytes, it is difficult to design specific sensing materials for each analyte.

To address the detection drawbacks of both monovariable sensors and sensor arrays in multi-analyte recognition, multivariable sensors employ multivariable transducers to interpret the differential responses of cross-sensitive materials, thereby increasing output variables [8, 83, 85]. This approach fundamentally differs from the sensor array configuration that relies on multiple sensing units [188, 197]. The implementation of multivariable sensing technology allows for multi-analyte recognition tasks using minimal or even a single sensing material, which substantially expands the application scenarios of chemical sensors.

Figure 4b depicts the two-dimensional output characteristic curves of a multivariable sensor with two output variables when performing three analytes identification. As illustrated by the blue dashed line, the three analytes generate superimposed responses on Output #1, yet they can be differentiated in the vertical direction by the introduction of Output #2. Figure 4c shows the output characteristic curves of four analytes in a three-dimensional sensing space. By expanding the number of output variables and increasing the dimensionality of the sensing space, the classification and identification of more kinds of analytes can be realized. It is crucial to acknowledge that the numerous output variables generated by a multivariable sensor must be independent of one another (e.g., orthogonal Output #1 and Output #2) [198].

Two principal methods exist for obtaining multiple output variables. The first entails altering the operational conditions of the sensor, including modifying the operating temperature, light conditions, and operating voltage, among other factors [3, 199–202]. The second involves extracting the desired variables from the sensor output characteristic curves. The output characteristic curves serve as a direct source for acquiring multivariable outputs, reflecting the property variations of sensors in the presence of analytes. For instance, noise spectra, utilized to evaluate the noise levels of sensors at different frequencies, contain rich sensing information due to their generation mechanisms being closely associated with physical processes on material surfaces and carrier scattering [203]. The time-dependent response curves under varying analyte concentrations represent the most intuitive and readily obtainable output, from which a series of feature variables characterizing the sensor’s dynamic properties can be extracted [204]. Finally, the FET transfer characteristic curves, obtained by scanning the gate voltage, influence the interaction between analytes and sensing materials through varying electric fields, thereby generating curves with distinct change patterns for multivariable extraction [205]. In the following section, these two predominant feature extraction schemes will be discussed through representative examples.

#### Feature Extraction Schemes: Changing Operational Conditions

The optimal operating temperature for a given analyte can be determined by considering the analyte molecular bond dissociation energies, adsorption modes, and molecular volumes [206]. Establishing a relationship between the optimal operating temperature and the analyte species can enhance the sensor’s selectivity and facilitate analyte identification [207]. This strategy is commonly employed in metal oxide semiconductor chemical sensors but is less prevalent in carbon-based chemical sensors, which can operate at room temperature.

Light modulation is a non-contact, low-power approach to material modification, capable of modulating low-dimensional carbon-based materials with large specific surface areas [208, 209]. It offers a straightforward and effective method for output variable acquisition. As illustrated in Fig. 4d, Park et al*.* employed a UV light-emitting diode to locally illuminate an unspecifically modified graphene field effect transistor (GFET) with the objective of modifying the electron transport properties of graphene, thereby enhancing the sensitivity and selectivity of the sensor to volatile compounds [54]. By comparing the resistance changes of the GFETs following exposure to ethanol, water, and dimethyl methylphosphonate (DMMP) in the absence and presence of UV illumination revealed that the sensitivities of the three gases were 54, 4.2, and 2, respectively. Furthermore, the changes in the response-time curves induced by illumination provided an additional dimension of gas information, enabling the detection of the three gases in a two-dimensional response plane with only one GFET. Temperature and light modulation are commonly employed in a variety of chemical sensors, but all necessitate additional heating and light apparatus for optimal functionality.

The distinctive three-electrode configuration of FET chemical sensors facilitates the regulation of channel carrier concentration through the application of a gate voltage, thereby offering a more convenient approach to the acquisition of output variables [208, 210]. As illustrated in Fig. 4e, Maity et al*.* employed helical polyaniline (PANI)@CNT as the channel material of the FET-type gas sensor. The channel was subjected to chiral modulation by applying a gate voltage, thereby enabling the recognition of the chiral molecule limonene (S( +)/R(-)) [211]. The distinctive cylindrical and curved configuration of CNTs enables them to exhibit heightened spin–orbit Rashba interactions in comparison to conventional two-dimensional planar materials. This property endows them with exceptional field-controlled chiral discrimination and chiral spintronics capabilities. The experimental results demonstrate that chiral molecules exhibit a preference for interacting with the sensor at specific gate voltages. Distinct from sensing mechanisms relying on the intrinsic material properties, gate voltage modulation primarily manipulates the energy band structure of sensing materials through electric field control. This modulation alters the Schottky barrier formed at the interface between the electrode and the sensing material, thereby regulating the sensor’s sensitivity [212]. Moreover, since the binding energy of analytes on the sensing material is strongly dependent on their adsorption orientation [213], the application of gate voltages with different polarities can modify the orientation of analytes, consequently enhancing the sensor’s desorption efficiency [120, 212, 214].

The control of heating, illumination and gate voltage represents straightforward and efficacious methods for the generation of multivariable outputs. In essence, these three approaches serve to enhance the strength of the interaction with a specific analyte, primarily by modifying the intrinsic properties of the sensing material (e.g., carrier transport properties, surface reactivity, and carrier spin–orbit states). Modifying sensor operating parameters represents an expedient and accessible approach to augmenting one-dimensional sensing information into higher dimensions. This methodology provides a solution for enhancing the sensor’s multi-analyte recognition capabilities and facilitating an understanding of underlying sensing mechanisms.

#### Feature Extraction Schemes: Extracting Characteristic from Output Curves

Obtaining multivariable outputs by changing multiple operating conditions of the sensor (e.g., simultaneously changing the operating temperature and light conditions) is a complex operation that increases the use cost in practical applications. However, extracting multivariable outputs from sensor output characteristic curves (noise spectra, response-time curves, transfer characteristic curves, etc.) can yield a large amount of high-dimensional sensing information without changing the sensor operating conditions.

As demonstrated in Fig. 4f, Rumyantsev et al. leveraged the low-frequency noise spectrum of a single graphene FET-type gas sensor to achieve selective detection of tetrahydrofuran, methanol, acetonitrile, and chloroform [215]. The experimental results demonstrate that the various gas molecules exert disparate effects on the low-frequency noise spectrum of graphene. Some gases induce alterations in the resistance of graphene, whereas others exert influence on the noise spectrum of graphene, introducing distinctive bumps on its 1/f background. It is interesting to note that the variations in graphene resistance and noise spectra are uncorrelated. The construction of high-dimensional sensing information from independent output variables is a crucial aspect in the recognition of multiple analytes by a single device. The extraction of multivariable outputs from noise spectra has been extensively employed for both chemoresistive and FET-type chemical sensors [215, 216]. However, its high requirements for test equipment restrict its implementation in portable detection devices.

As illustrated in Fig. 4g, Huang et al*.* utilized eleven output variables derived from the response-time curves of a single CuPc-modified graphene FET-type gas sensor for the detection of NH_3_ and PH_3_ [217]. The eleven extracted output variables obtained from the exposure and flushing phases of the response-time curves were subjected to PCA and LDA pattern recognition algorithms for the identification of gas concentration and species. The experimental results demonstrate that the accuracy, sensitivity, and specificity of the sensor for both gases are nearly 100% at the 500 ppb level. The application of time-dependent response curves for the extraction of multivariable outputs is most prevalent in chemoresistive sensors, where the test is straightforward and the number of output variables that can be extracted is considerable. However, the output variables obtained through this extraction method are challenging to correlate with the underlying sensing mechanisms, and it is not feasible to achieve sensing mechanism-oriented output variable extraction.

FETs have a variety of output signals, including time-dependent *I*_ds_, output characteristic curves, and transfer characteristic curves. Among these, the transfer characteristic curve represents the relationship between the measured *I*_ds_ and *V*_gs_ at a fixed *V*_ds_. The rich variation patterns of the transfer characteristic curve in the analytes not only contain valuable sensing information for identification but also reveal the underlying sensing mechanisms. Figure 4h illustrates the four distinct variation patterns of transfer characteristic curves observed in CNT-based FET-type biosensors exposed to biomarkers. These variation patterns illustrate the underlying sensing mechanisms, which include electrostatic gating, Schottky barrier modulation, gate capacitance modulation, and carrier mobility modulation [218]. Electrostatic gating describes a process wherein adsorbed analytes modulate the local electric field or carrier concentration of CNTs through electrostatic interactions, resulting in a leftward or rightward shift of the transfer characteristic curves [219]. Since the interaction is mediated solely by electrostatic forces, it does not involve direct charge transfer [220]. The Schottky barrier modulation is attributed to the adsorption of biomarkers at the electrode-CNT contact, which alters the energy band alignment by modifying the local work function [221]. The Schottky barriers exert disparate inhibitory effects on the transport of holes and electrons, thereby influencing the p- and n-branches of the transfer characteristic curves to diverge in opposing directions. Gate capacitance modulation is attributable to adsorbed low permittivity biomarkers, which exert an effect on the gate capacitance and, as a consequence, modify the gate voltage control efficiency [222]. Consequently, the p- and n-branches of the transfer characteristic curve are tilted to the left and right, respectively. Carrier mobility modulation refers to the scattering effect of adsorbed biomarkers on carriers, thus affecting the carrier mobility [223]. This results in a simultaneous change to the conductance of the p- and n-branch of the transfer characteristic curve. The four curve variation patterns illustrated in Fig. 4h provide a qualitative basis for analyzing the sensing mechanism. By extracting the output variables that reflect the subtle changes in the curves, it is possible to achieve the identification of analytes.

In graphene-based FET-type chemical sensors, the left/right shift of the Dirac point is employed extensively for analyte differentiation identification [224]. Figure 4i illustrates a schematic of the differentiation between positive and negative samples of COVD-19, utilizing Dirac point offsets [225]. This research demonstrated 100% detection accuracy for ten positive and ten negative samples using unmodified crumpled graphene. The use of the Dirac point offset as the sole output variable presents a significant challenge in the detection of multiple analytes in complex chemical atmospheres.

Similar to Fig. 4h, graphene-based chemical sensors are also capable of reflecting the underlying sensing mechanisms through the curve variation patterns, which provides a good basis for the analyte identification. Figure 4j illustrates four variation patterns of transfer characteristic curves for graphene-based FET-type chemical sensors, corresponding to four sensing mechanisms [131]. Firstly, the transfer of positive and negative charges between the analyte and graphene results in an overall left/right shift of the curve, namely a left/right shift of the Dirac point. Secondly, the charged analyte affects the hole mobility through the Coulomb force, which causes a change in the slope of the p-branch. Thirdly, similarly, electron mobility is affected by charged analytes, causing a change in the slope of the n-branch. Finally, the upward/downward shifts of the Dirac point, which is the residual carrier to charged impurity concentration ratio, can also be affected by the charged analyte. The distinctive V-shaped transfer characteristic curve of graphene displays diverse variation patterns in analytes. By extracting multiple output variables that can characterize the curve variations, a multi-dimensional sensing information space is established, enabling the final analyte identification.

Figure 4h-j illustrates the distinct transfer characteristic curves of carbon-based FETs in the presence of different analytes, where the extraction of multiple output variables that quantitatively describe the curve variations serves as a prerequisite for analyte identification. Three primary methods are typically employed for parameter extraction: 1) intrinsic electrical parameters of FETs, 2) drain-source current (*I*_ds_) at various gate voltages (*V*_gs_), and 3) composite parameters. Firstly, the intrinsic electrical parameters of FETs include threshold voltage (*V*_th_), transconductance (*g*_m_), carrier mobility (*μ*), subthreshold swing (*SS*), current on/off ratio (*I*_on_/*I*_off_), and saturation current. The *V*_th_, which characterizes the gate voltage at which the FET transitions from the off-state to the on-state, can be extracted using methods such as the maximum transconductance method or linear extrapolation technique [226]. This parameter effectively evaluates the overall lateral shift of the curves. The *g*_m_, defined as *g*_m_ = (∂*I*_ds_/∂*V*_gs_)|*V*_ds_ = const, quantitatively describes the amplification capability of the FETs and can be used to assess the slope of the p/n branch [130]. The *μ*, which characterizes the ability of charge carriers to move under an applied electric field, is calculated as *μ* = (*L*_c_/*W*_c_*V*_ds_*C*_ox_)∙(∂*I*_ds_/∂*V*_gs_), where *L*_c_, *W*_c_, and *C*_ox_ represent the channel length, channel width, and gate oxide capacitance per unit area, respectively. This parameter serves as a quantitative descriptor of analyte-induced modifications in the properties of the sensing material [227, 228]. Although *SS*, *I*_on_/*I*_off_, and saturation current can also quantitatively describe curve variations, they are less frequently employed. Secondly, to achieve a more detailed characterization of curve variations, the *I*_ds_ at different *V*_gs_ can be extracted. This method is straightforward and reliable, requiring no complex calculations [229]. Thirdly, composite parameters are derived from combinations of multiple variables or functions through mathematical operations or transformations. Examples include the ratio of the remaining carrier concentration to the concentration of charged impurities (n^*^/*n*_imp_), the ratio of electron to hole carrier mobility (*µ*_*e*_/*µ*_*h*_), and the product of carrier mobility and threshold voltage (*µV*_th_). Well-designed composite parameters not only facilitate multi-analyte identification but also provide quantitative insights into sensing mechanisms [131, 132].

## Carbon-Based Field-Effect Transistor-Type Chemical Sensors for Multivariable Sensing Applications

The distinctive variation patterns of transfer characteristic curves of carbon-based FET-type chemical sensors in analytes not only elucidate the underlying sensing mechanisms but also facilitate the extraction of pivotal electrical parameters as output variables for analyte identification. This section presents methodologies for the extraction electrical parameters from transfer characteristic curves, as well as discuss their applications in combination with pattern recognition algorithms for classification and recognition.

### Feature Extraction and Multivariable Sensing Applications of CNTs-Based FETs

**Fig. 5:**
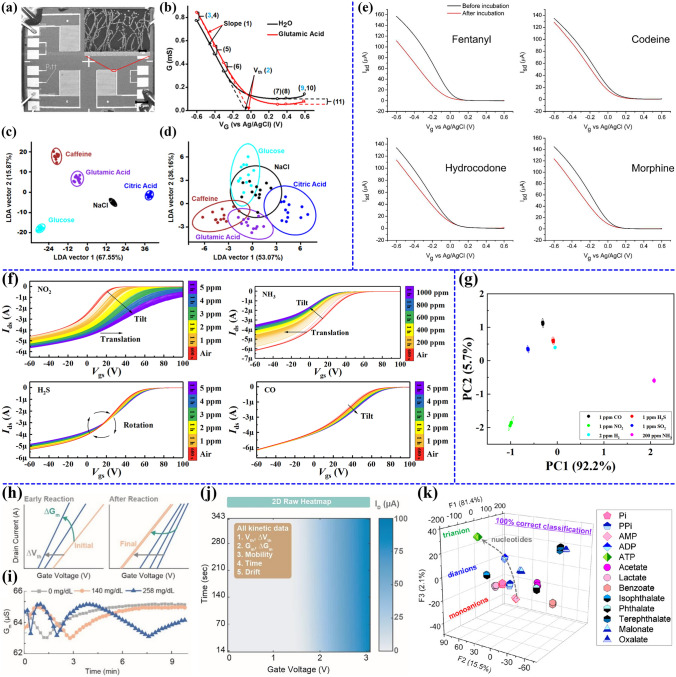
**a** SEM images of the carbon nanotube-based FET-type sensor array and channel region. **b** Eleven feature parameters extracted from the variation of transfer characteristic curves. Classification results of five basic tastants performed by LDA using **c** eleven and **d** three feature parameters. Reproduced with permission [229]. Copyright 2017, American Chemical Society. **e** Variation of transfer characteristic curves of carbon nanotube-based FET-type sensors in fentanyl, codeine, hydrocodone, and morphine. Reproduced under terms of the CC-BY license [231]. Copyright 2024, Wiley–VCH. **f** Variation of transfer characteristic curves of carbon nanotube-based FET-type sensors in four atmospheric pollutants including tilt, translation and rotation. **g** Classification results of six atmospheric pollutants using eight feature parameters combined with PCA. Reproduced with permission [130]. Copyright 2024, American Chemical Society. **h** Diagram illustrating the changes in the transfer characteristic curve at various stages of the enzymatic reaction. **i** Typical G_m_ variation during the enzymatic reaction. **j** Time-evolved transfer characteristic curves displayed as a 2D current heatmap. **h-j** reproduced with permission [49]. Copyright 2024, American Chemical Society. **k** Qualitative analysis of 13 oxyanions (500 μM) using LDA, achieving 100% correct classification. Reproduced with permission [235]. Copyright 2022, American Chemical Society

Unlike graphene, the transfer characteristic curves of CNT-based FET-type chemical sensors do not usually have a distinct bipolar character. Therefore, output variable extraction is usually performed by utilizing the curve changes induced by one type of carrier, e.g., p-branch (holes) or n-branch (electrons) curve changes. Silva et al*.* used metal nanoparticle-modified CNT-based FET-type sensor arrays (bare-oxidized SWCNT, Au, Pt, Rh, and Pd) to classify five basic tastants: saltiness (NaCl), sweetness (glucose), sourness (citric acid), bitterness (caffeine), and umami (glutamic acid) [229]. Figure 5a depicts the SEM images of the sensor array and the channel area (inset). Figure 5b illustrates the method of extracting eleven features from the transfer characteristic curve. The meanings of each parameter are as follows: (1) relative change in transconductance, (2) threshold voltage (*V*_th_) shift, (3, 10) relative change in conductivity at ± 0.6 *V*_g_, (4–9) change in overall conductance normalized to conductance at *V*_th_, and (11) the relative change in minimum conductance. The eleven features were input into the LDA algorithm as output variables of the sensor array for the differentiation of the five analytes. The results of the differentiation without overlap in two dimensions are shown in Fig. 5c. In contrast, when only three features (minimum transconductance, maximum transconductance, and threshold voltage) were utilized, the classification outcomes of the five analytes overlapped (Fig. 5d), indicating that an insufficient number of features can impede the identification of alterations in the curve. In subsequent work, Bian et al. adopted the same feature extraction scheme to successfully differentiate five purine compounds (adenine, guanine, xanthine, uric acid, and caffeine) [230]. They found that employing SVM could reduce the number of required feature variables while maintaining almost the same classification accuracy. Furthermore, the interactions between structurally similar purine derivatives and the sensor were analyzed using density functional theory (DFT) calculations. These works employ the multivariable output capacity of carbon-based chemical sensors to develop a solution for achieving analyte identification with non-specifically modified sensor arrays. The employed feature extraction scheme is an effective means of accurately portraying the curve variation. Shao et al*.* employed Au nanoparticles to modify a CNT-based FET-type sensor for the recognition of four classes of opioids [231]. Figure 5e illustrates the variation of transfer characteristic curves in fentanyl, codeine, hydrocodone, and morphine. To extract sensing information from the curves with a similar variation pattern, the authors extracted fifteen feature parameters as inputs to the LDA, achieving a recognition accuracy of 91.2% for the four opioids. To improve the detection of trace fentanyl (less than 10 ng mL^−1^), the fentanyl antibodies were immobilized on the surface of gold nanoparticles, resulting in a LoD of 10.8 fg mL^−1^. In this work, the sensor array, composed of both specific and non-specific sensing units, is employed for the recognition of multiple analytes and the extension of the sensor’s linear range.

To augment the number of analyte species that can be identified and to diminish the number of sensing units in the array, Shi et al*.* employed a Pd-modified CNTs random network as the channel of a FET-type gas sensor for the identification of six atmospheric pollutants [130]. In this study, three types of gas adsorption sites were constructed: the homogeneous CNTs random network, Pd nanoparticles deposited on the CNTs surface, and the source-drain electrodes composed of Pd/Au. These provide different pathways for analytes with different chemical properties to interact with the sensor. Figure 5f illustrates the variation patterns of the transfer characteristic curves in four gases: tilt, translation, and rotation. From these curves, eight feature parameters were extracted: Δ*g*_m_, Δ*V*_th_, Δ*I*_ds(−60V)_, Δ*I*_ds(−40V)_, Δ*I*_ds(−20V)_, Δ*I*_ds(0V)_, Δ*I*_ds(20V)_, and Δ*I*_ds(40V)_. The parameter Δ*g*_m_ is used to quantify the tilt of the curve, which represents the change in carrier mobility. Similarly, the parameter Δ*V*_th_ is employed to describe the curve’s left–right translation, which corresponds to the alteration in carrier concentration. Additionally, the remaining Δ*I*_ds_ at varying gate voltages are utilized to illustrate the curve’s variation trend in greater detail. Figure 5g illustrates the outcomes of the classification process conducted using PCA, wherein all six gases were successfully distinguished from one another, exhibiting no overlap. This work essentially analyzes the influences of gas molecules on the transfer characteristic curves and establishes the correlation between electrical parameters and gases with different chemical properties. The classification and identification of six gases by one sensor is realized, overcoming the problem of many sensing units and large size required for the sensor array.

Multivariable sensing technology has also been widely applied in biosensors, with the majority of applications focusing on distinguishing target analytes from interfering substances. This is particularly critical because many biological samples (e.g., blood, sweat, saliva, and urine) contain not only the target analytes but also various substances that can influence sensing results [232]. In the works by Bian et al. and Liu et al., CNT-based FET-type sensors were used for the detection of Hg^2^⁺ [205], live/dead cells [233], and bacterial vaginosis (BV) positive/negative samples [234]. In these studies, the authors extracted features from the transfer characteristic curves and, by combining pattern recognition algorithms (e.g., random forest, LDA, SVM, and PCA), optimized the feature parameters to achieve highly selective detection of the target analytes. Jang et al. integrated a paper-based analytical cartridge with a FET and analyzed the kinetic data from transfer characteristic curves using a deep learning model, successfully performing quantitative analysis of cholesterol concentrations in patient plasma samples [49]. As shown in Fig. 5h, conventional FET analysis methods typically focus on a single output variable (e.g., ΔV_th_) and the states at the initial and end points, which fail to capture changes occurring during enzyme reactions that are influenced by time-dependent enzymatic reaction rates and sample matrix effects. In Fig. 5i, the changes in G_m_ during enzymatic reactions are displayed, reflecting the effects of enzymatic reaction rates, mixing processes in the cartridge, and sample matrix effects. To comprehensively capture and interpret the dynamic nature of these biochemical processes, the authors leveraged the universal function approximation capability of neural networks to analyze the heatmap of transfer characteristic curves shown in Fig. 5g. This heatmap incorporates all the enzymatic kinetics details, such as V_th_, ΔV_th_, G_m_, ΔG_m_, mobility, time, and drift. Through deep learning-based analysis of these feature parameters, detection of proteins in plasma samples was achieved with minimal interference from varying pH levels in plasma, a coefficient of variation (CV) as low as 6.46%, and an r^2^ greater than 0.976. Mitobe et al. developed an organic FET biosensor with an extended-gate structure [235]. By analyzing the output characteristic curves using LDA and SVM, they achieved the detection of 13 oxygen-containing anions with a single sensor and demonstrated the highly selective analysis of hydrogen monophosphate present in human serum. These studies collectively highlight the immense potential of multivariable sensing technology in biosensing applications. By leveraging the high-dimensional sensing space constructed from multiple output variables, it is possible not only to achieve multi-analyte recognition but also to eliminate the influence of interfering analytes, thereby improving selectivity.

### Bipolar Curve Feature Analysis for Enhanced Analyte Recognition of Graphene-Based FETs

**Fig. 6:**
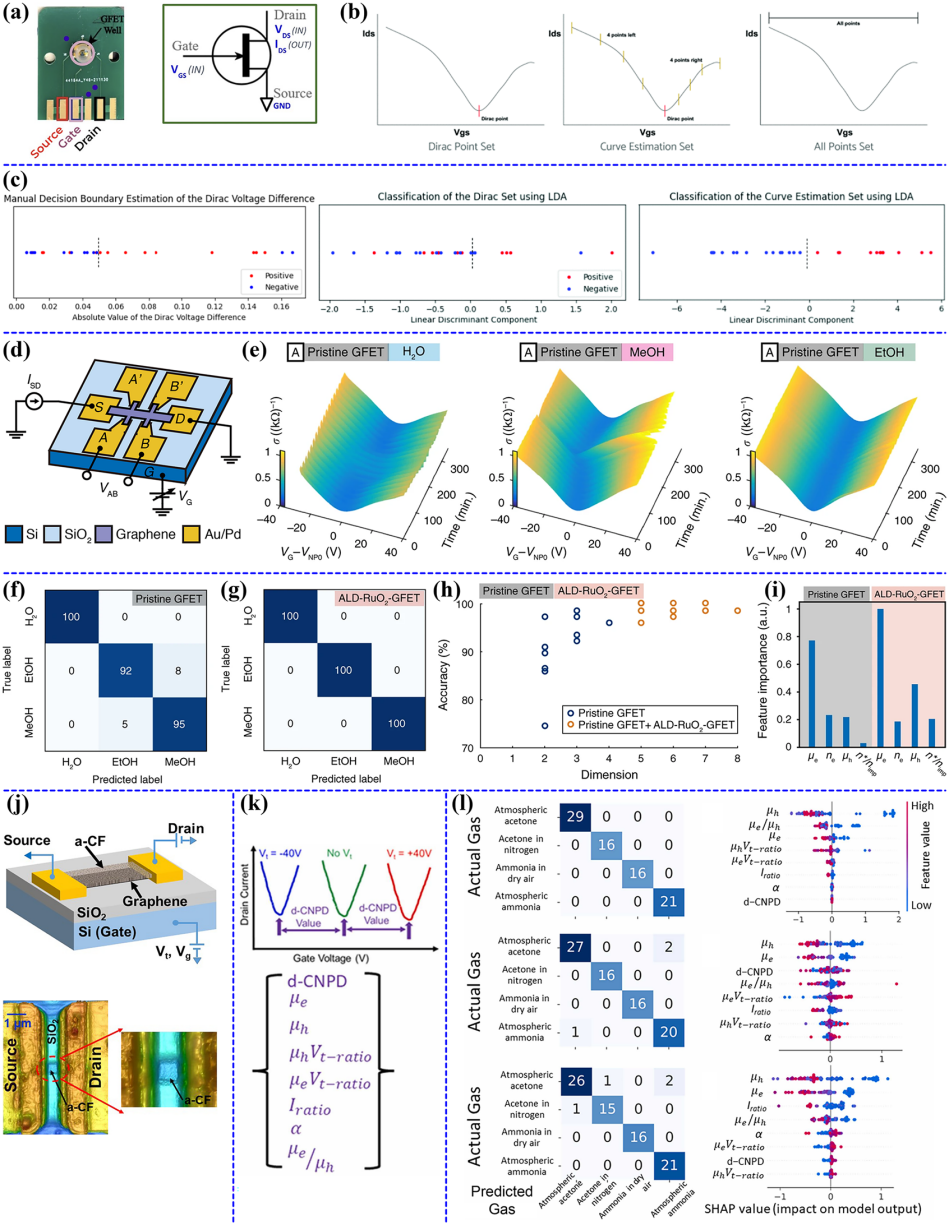
**a** Optical photograph and schematic of graphene-based FET-type biosensor. **b** Three transfer characteristic curve feature extraction schemes: the Dirac Point Set, the Curve Estimation Set, and the All Points Set. **c** Manual decision boundary estimation of the Dirac voltage difference, classification of the Dirac Point Set using LDA, and classification of the Curve Estimation Set using LDA. Reproduced with permission [237]. Copyright 2023, IEEE. **d** Schematic diagram of a graphene-based FET-type gas sensor. **e** Transient conductivity distribution of an unmodified graphene sensor (Pristine GFET) in three analytes as a function of gate voltage. Confusion matrices for prediction using a multilayer perceptron classifier with a feed-forward neural network structure: **f** Pristine GFET, **g** ALD-RuO_2_-GFET. **h** Accuracy of predictions using different number of feature parameters. **i** Results of feature importance studies performed using one-way analysis of variance (ANOVA) F-test. Reproduced under terms of the CC-BY license [131]. Copyright 2020, Nature Publishing Group. **j** Schematic and laser microscopy images of the channel region of a graphene-based FET-type gas sensor functionalized using activated carbon (a-CF). **k** Schematic of transfer characteristic curve test using Charge Neutrality Point Disparity (CNPD) and eight feature parameters extracted from it. **l** Confusion matrices for four analytes identified using XGBoost, KNN and Naïve Bayes models. Importance of feature parameters obtained using Game theory approach of SHapley Additive exPlanations (SHAP). Reproduced with permission [132]. Copyright 2023, Elsevier

The distinctive V-shaped transfer characteristic curve and exceptional monomolecular sensitivity of graphene have contributed to its extensive utilization in multivariable chemical sensors. Researchers have devised a multitude of techniques for extracting features as sensor output variables, based on the characteristics of the curve and the underlying sensing mechanisms. Sensi et al. designed an ambipolar electrolyte-gated transistor immunosensor (rGO-EGT) using rGO as the channel and an infliximab (IFX)-specific probe functionalized on the gate electrode [236]. This sensor enables the selective and quantitative detection of anti-drug antibodies (ADAs). The concentration of IFX antibodies (ATI) in patient serum is typically very low (1–10 pM), and detection is often hindered by interference from tumor necrosis factor-alpha (TNF-α). The authors first employed the Gumbel distribution to fit the transfer characteristic curves, from which they extracted three key parameters: *V*_cnp_, *I*_cnp_, and *α*, corresponding to the voltage, current, and curvature parameters at the Dirac point, respectively. These parameters were used to quantitatively analyze the changes in the transfer characteristic curves of the rGO-EGT in the presence of analytes. The sensor not only effectively distinguished ATI from TNF-α but also achieved a theoretical detection limit for ATI as low as 10 aM. Figure 6a illustrates the graphene-based FET-type biosensor developed by Tsui et al*.* [237]. The identification of COVID-19 positive and negative samples was conducted using the three curve feature extraction schemes depicted in Fig. 6b in conjunction with LDA, SVM, and PCA pattern recognition algorithms. The Dirac Point Set comprises four dimensions: the voltage and current at the baseline Dirac point, and the voltage and current at the sample Dirac point. The Curve Estimation Set contains nine dimensions: the Dirac point, four uniformly spaced I-V points to the left of the Dirac point, and four uniformly spaced I-V points to the right. The All Points Set employs all points in the curve to construct the feature space. Figure 6c illustrates the outcomes of the classification process utilizing the manual decision boundary delineated by the Dirac Point Offset, with an accuracy rate of 68.4%. There is considerable overlap in the classification results obtained using the Dirac Point Set in conjunction with LDA, with an accuracy of 65.7%. By using the higher dimensional Curve Estimation Set and All Points Set to depict the curve variations in more detail, with the support of the LDA algorithm, the accuracy is 96.2% and 99.0%, respectively. It can be observed that the utilization of higher-dimensional feature spaces facilitates enhanced recognition. However, an excess of features yields limited improvement and increases the computational burden of the sensing system. The sensing mechanism should be employed as a guiding principle to extract critical feature parameters as the output variables of the sensor.

Figure 6d illustrates a graphene-based FET-type gas sensor developed by Hayasaka et al. The transfer characteristic curve is composed of four distinctive physical attributes, forming four-dimensional output variables. This enables precise quantitative classification of water, methanol, and ethanol, as well as qualitative differentiation of binary mixtures [131]. Figure 6e illustrates the transient conductivity distribution of the unmodified graphene sensor (Pristine GFET) in three analytes as a function of gate voltage. In accordance with the four gas detection mechanisms illustrated in Fig. 4j, the V-shaped conductivity curves are decomposed into four distinct physical parameters: carrier concentration (*n*_e/h_), hole mobility (*µ*_h_), electron mobility (*µ*), and the ratio of the remaining carrier concentration to the concentration of charged impurities (n^*^/*n*_imp_). Information specific to the gas molecules on the graphene surface, such as charge magnitude, dipole moment, etc., is stored through these physical parameters [238–240]. Figure 6f-g illustrates the confusion matrices for predictions generated by a multilayer perceptron classifier with a feed-forward neural network structure. The Pristine GFET exhibits an accuracy of 96.2%, whereas another sensor with RuO_2_ functionalization via ALD (ALD-RuO_2_-GFET) demonstrates an accuracy of 100%. Figure 6h shows the classification accuracy when using any two or three of the four pristine GFET features (*n*_e/h_, *µ*_e_, *µ*_h_, and n^*^/*n*_imp_), as well as the classification accuracy when the ALD-RuO_2_-GFET features are included. It can be observed that extending the feature space to higher dimensions results in improved accuracy. Figure 6i depicts the results of the feature importance study, which was conducted using a one-way analysis of variance (ANOVA) F-test. The results indicate that *µ*_e_ is the most important feature, while n^*^/*n*_imp_ is the least important. This work presents a novel approach to multi-analyte identification, whereby key physical parameters that reflect the sensing mechanism are extracted from transfer characteristic curves. This method avoids the addition of multiple functional materials, offering a robust theoretical and experimental foundation for the development of miniaturized sensor arrays.

In addition to the doping and scattering induced by gas adsorption, Agbonlahor et al*.* demonstrated that the distinctive charge transfer resulting from the formation of van der Waals (vdW) bonding (i.e., graphene-molecule vdW complexes) between adsorbed gases and graphene channels can also be utilized as a means of gas recognition [132]. Figure 6j illustrates a schematic of the activated carbon (a-CF) functionalized graphene-based FET gas sensor, accompanied by a laser microscopy image of the channel region. This work introduces a Charge Neutrality Point Disparity (CNPD) test scheme. The method employs a voltage (*V*_t_) that modulates the strength of the vdW interaction between graphene and adsorbed gas molecules, applied via the gate before the transfer characteristic curve measurement. Figure 6k illustrates a schematic of the transfer characteristic curve test utilizing CNPD, along with the eight feature parameters extracted from it. The d-CNPD monitors the charge transfer induced by *V*_t_ modulated gas adsorption. The *µ*_*e*_ and *µ*_*h*_ are used to detect electron and hole scattering, respectively. The *µ*_*h*_*V*_*t-ratio*_ and *µ*_*e*_*V*_*t-ratio*_ are employed to assess the impact of *V*_*t*_ polarity on graphene-gas hole/electron, respectively. The *I*_*ration*_ and α are utilized to monitor the *I*_ds_ alterations. The *µ*_*e*_/*µ*_*h*_ is utilized to assess the relative changes in electron and hole mobility. Figure 6l illustrates the confusion matrices for the identification of atmospheric ammonia, atmospheric acetone, ammonia in dry air, and acetone in nitrogen using XGBoost, KNN, and Naïve Bayes models. The respective accuracies are 100%, 96.34%, and 95.12%. The significance of the feature parameters obtained using the Game theory approach of SHapley Additive exPlanations (SHAP) is shown in Fig. 6l, revealing the correlation between the feature parameters and the accurate model output predictions. In this study, the researchers concentrated on the doping and scattering characteristics of graphene-molecule van der Waals complexes. These were derived as feature variables that reflected the properties of the analytes through CNPD testing, which ultimately resulted in the precise identification of four analytes using a single sensor.

Whether it is a CNT or a graphene-based FET-type chemical sensors, the rich transfer characteristic curves in the analytes are the key to their multivariable sensing capability. Various feature parameter extraction schemes are directed to the purpose of establishing one-to-one fingerprint information between analytes and curve variation patterns by extracting key parameters that are closely related to the sensing mechanism. Overcoming the complex specificity modification process of the sensor array, the qualitative and quantitative analysis of multiple analytes using a few or even a single sensor is realized.

## Conclusion and Outlook

In this review, research progress of carbon-based multivariable chemical sensors used for the recognition of multiple analytes in complex chemical environments was reviewed. From the earliest monovariable chemical sensors to sensor arrays to multivariable chemical sensors, the evolution of these technologies reflects researchers’ continuous efforts to develop high-performance, multifunctional, and miniaturized sensing devices. The rapid developments in the sensing field have resulted in the emergence of a large number of sensing materials with excellent properties and converters with various structures. How to fully exploit the variations in the properties of the sensing material and the output variables of the transducer will be critical to achieving further progress in the field. The design of multivariable chemical sensors is based on three fundamental criteria: 1) the use of sensing materials that can respond differently to analytes; 2) the incorporation of transducers that are able to read and translate changes in the properties of the sensing materials into multiple independent output variables; and 3) the integration of pattern recognition algorithms that are capable of analyzing the data and providing identification results [8]. The large specific surface area, monomolecular detection capability, and diverse modes of interaction with analytes of carbon-based materials such as graphene and CNTs make them ideal as sensing materials for multivariable sensors. The unique amplification capability and transfer characteristic curve of FETs that varies with the external chemical environment make them capable of multivariable outputs. The combination of carbon-based materials and FETs has reinvigorated the development of multivariable sensors. With the in-depth study of carbon-based FET-type chemical sensors, a variety of schemes for extracting output variables from transfer characteristic curves have been developed. From the simple extraction of feature points in the curve to the decoupling of physical parameters from the curve that can reflect the sensing mechanisms, it has been shown that the establishment of fingerprint information between analytes and output variables needs to be oriented to the comprehensive understanding of the sensing mechanisms. The advancement of computer science has led to the advent of numerous high-performance pattern recognition algorithms, which have undoubtedly served as a catalyst for the development of multivariable sensor output variable extraction and analyte identification.

The multianalyte recognition capability of carbon-based multivariable sensors enables them to provide excellent selectivity and stability in various types of complex chemical environments, such as the detection of blood/sweat biomarkers, atmospheric pollutants, and toxic industrial chemicals. To further develop the application potential of multivariable sensors, it is essential to explore the following aspects.

Firstly, the root of multivariable output capability is attributed to the diverse sensing mechanisms between the sensing material and the analyte. Bottom-up theoretical computational tools facilitate an exhaustive, quantitative comprehension of the impact of analyte species and concentration on material properties [241–243], including temperature, conductivity, work function, and permittivity. This understanding can guide the design of novel sensing materials as well as the modification of existing materials. In recent years, the development of hybrid materials, particularly those combining carbon with other 2D materials (e.g., graphene-MoS_2_ heterostructures), has emerged as a promising trend [244–246]. These hybrid materials leverage the synergistic effects of their constituent components, enabling tailored electronic properties, improved interfacial interactions, and enhanced sensitivity [247]. Such advancements are pivotal for achieving sensing materials with monomolecular sensitivity and diverse response modes, driving the evolution of multivariable sensors. Furthermore, the development of pre-positioned molecular filters that are integrated with sensing materials will enhance the stability and selectivity of the sensors in challenging chemical environments [248, 249], such as those containing potent interfering analytes and high humidity. Complementing this, advanced materials like tailored nanomaterials and polymer composites [250–252], as well as hybrid sensing platforms combining multiple transduction mechanisms (e.g., optical, electrochemical, and thermal) [253–255], further enhance selectivity and reduce overlap, improving sensor reliability.

Secondly, the FET must be capable of accurately converting the input signal from the sensing material, while accounting for any output bias introduced by external factors. The sensing material is in direct contact with the dielectric layer of the FET, and the complex interfacial environment affects the carrier transport in the sensing material thereby generating an output drift, e.g., carrier fluctuation due to surface defects and dangling bonds. The optimization of the dielectric layer preparation process and the implementation of surface treatment can enhance the output stability of FET devices. Furthermore, the testing of transfer characteristic curves necessitates the continuous scanning of the gate voltage. However, prolonged gate bias may result in the fixation of charged analytes on defects in the dielectric layer. This will offset some of the gate voltage, causing a threshold voltage drift [256]. The development of novel electrical test methods, such as pulsed gate voltage scanning, can reduce the impact on the intrinsic electrical performances of FETs [257, 258].

Thirdly, the extraction and screening of output variables from multivariable sensors, as well as the analysis of high-dimensional sensing data, necessitates the utilization of high-performance pattern recognition algorithms that can be directly integrated into sensor systems to enable real-time processing and interpretation of complex data streams. Given the constrained computational resources of miniaturized sensing devices, future pattern recognition algorithms will need to pre-screen the “most valuable” items from a large number of output variables in order to improve the efficiency of analyte identification. To this end, more efficient algorithms, such as sparse representation techniques [259, 260] or feature selection methods based on mutual information [261–263] or PCA, can be employed to extract independent and informative features from the output variables. These methods reduce redundancy and dimensionality, thereby optimizing computational efficiency without compromising analytical performance. Furthermore, it is challenging to gather a substantial amount of experimental data for the model training of pattern recognition algorithms within a limited time. Developing pattern recognition algorithms applicable to a small number of samples will reduce the difficulty of obtaining experimental data and improve development efficiency.

In conclusion, the integration of carbon-based materials with FETs represents a significant step forward in the practical application of multivariable sensors for analyte identification. Looking ahead, further advancements in sensing materials, transducers, and pattern recognition algorithms are poised to usher in a new era of highly efficient and versatile sensors. Beyond their technical potential, these innovations promise transformative societal impacts. In environmental monitoring, such sensors could enable real-time, precise detection of pollutants, driving more effective conservation and public health strategies. In healthcare, they may revolutionize early disease diagnosis, personalized medicine, and continuous health monitoring through wearable or implantable devices. As these technologies evolve, their integration with artificial intelligence and data analytics could further enhance their predictive and diagnostic capabilities. Ultimately, the continued development of chemical sensing technologies holds immense promise for addressing pressing global challenges, fostering a healthier and more sustainable future.
